# Two-step conversion of polyethylene into recombinant proteins using a microbial platform

**DOI:** 10.1186/s12934-023-02220-0

**Published:** 2023-10-17

**Authors:** Alexander Connor, Jessica V. Lamb, Massimiliano Delferro, Mattheos Koffas, R. Helen Zha

**Affiliations:** 1 Department of Chemical and Biological Engineering, Rensselaer Polytechnic Institute, Troy, NY 12180 USA; 2 Center for Biotechnology and Interdisciplinary Studies, Rensselaer Polytechnic Institute, Troy, NY 12180 USA; 3 Chemical Sciences and Engineering Division, Argonne National Laboratory, 9700 S Cass Ave, Lemont, IL 60439 USA

**Keywords:** Microbial upcycling, Recombinant silk, Plastic waste, Sustainability, Synthetic biology

## Abstract

**Background:**

The increasing prevalence of plastic waste combined with the inefficiencies of mechanical recycling has inspired interest in processes that can convert these waste streams into value-added biomaterials. To date, the microbial conversion of plastic substrates into biomaterials has been predominantly limited to polyhydroxyalkanoates production. Expanding the capabilities of these microbial conversion platforms to include a greater diversity of products generated from plastic waste streams can serve to promote the adoption of these technologies at a larger scale and encourage a more sustainable materials economy.

**Results:**

Herein, we report the development of a new strain of *Pseudomonas* bacteria capable of converting depolymerized polyethylene into high value bespoke recombinant protein products. Using hexadecane, a proxy for depolymerized polyethylene, as a sole carbon nutrient source, we optimized media compositions that facilitate robust biomass growth above 1 × 10^9^ cfu/ml, with results suggesting the benefits of lower hydrocarbon concentrations and the use of NH_4_Cl as a nitrogen source. We genomically integrated recombinant genes for green fluorescent protein and spider dragline-inspired silk protein, and we showed their expression in *Pseudomonas aeruginosa*, reaching titers of approximately 10 mg/L when hexadecane was used as the sole carbon source. Lastly, we demonstrated that chemically depolymerized polyethylene, comprised of a mixture of branched and unbranched alkanes, could be converted into silk protein by *Pseudomonas aeruginosa* at titers of 11.3 ± 1.1 mg/L.

**Conclusion:**

This work demonstrates a microbial platform for the conversion of a both alkanes and plastic-derived substrates to recombinant, protein-based materials. The findings in this work can serve as a basis for future endeavors seeking to upcycle recalcitrant plastic wastes into value-added recombinant proteins.

**Supplementary Information:**

The online version contains supplementary material available at 10.1186/s12934-023-02220-0.

## Introduction

The overwhelming majority of plastics used today are derived from non-renewable petrochemical feedstocks and are considered “recalcitrant”, meaning they are not readily degraded in natural environments. Commodity plastics produced in high volumes, such as polyethylene and polypropylene, can take upwards of a thousand years to biodegrade [1]. Ubiquitous macro and microplastic debris physically and chemically harm wildlife ecosystems and have been demonstrated to harm human health as well [2–7]. Unfortunately, plastic manufacturing continues to grow annually, with over 350 million tons produced in 2020 alone. Packaging materials dominate the consumer plastics market, and nearly 40% of plastics used for packaging are landfilled at their end-of-life, with another 32% leaking into the environment [8]. Polyethylene is particularly problematic, as it is commonly found in single-use applications. Resultingly, polyethylene is the most commonly produced plastic, representing 30% of all plastics production [9]. Likewise, polyethylene is the most predominant plastic pollutant, accounting for 34% of all plastic pollution [10]. Despite existing recycling infrastructure, only 9% of polyethylene is recycled using primary or secondary (i.e. mechanical) recycling methods [11]. Mechanical recycling is inefficient, typically yielding materials that have inferior mechanical properties compared to virgin plastics [10]. Moreover, the cost of recycled plastics cannot compete with the low cost of virgin plastics [12]. Tertiary recycling strategies, such as pyrolysis or chemical depolymerization, produce low molecular weight chemicals, fuels, and even monomers that can be re-polymerized, thus offering potential advantages over mechanical recycling in a circular economy. However, industrial-scale tertiary recycling has been limited to date due to engineering and economic challenges [13].

Ultimately, decreasing the environmental impact of the plastics industry requires adoption of non-recalcitrant plastics produced by green synthesis methods. Moreover, developing strategies for valorizing existing recalcitrant waste is an important step in reducing the carbon footprint and pollution potential of the current “take-make-waste” linear plastic economy. Thus, there is a growing interest in technologies that utilize plastic waste as a feedstock to generate value-added materials (which may or may not share chemical similarity with plastics) [14, 15]. Often referred to as upcycling, these methods target products that are more sustainable than traditional plastics or which offer higher economic value versus recycled plastics [15, 16]. Abiotic methods demonstrated for upcycling polyethylene include catalytic depolymerization and pyrolysis [17–19]. However, these methods are limited in the composition of upcycled product generated, typically yielding hydrocarbons similar to wax or diesel fuel [17–19]. These methods may also be energy intensive, often requiring high heat, and use heavy metal catalysts.

Consequently, upcycling methods that utilize biological systems have gained research interest [16], for example, in the development of enzymes that hydrolyze poly (ethylene terephthalate) [20, 21]. Nevertheless, there is a limited number of works demonstrating successful biological upcycling of polyolefins such as polyethylene. In this context, polyethylene has been depolymerized into a distribution of smaller hydrocarbons (often rich in alkanes) that are subsequently used by various microbial strains as a carbon source [16, 22–24]. Strains grown on polyethylene-derived feedstock have been shown to produce endogenous biopolymers, polyhydroxyalkanoates (PHAs), demonstrating the potential upcycling of polyethylene directly into a useful biodegradable plastic [16, 22–24]. Until now, microbial upcycling of polyethylene has been largely limited to PHAs as the target product [16]. While PHAs can be used as a biodegradable plastic in single-use applications, their commercial adoption in this space is impaired by a relatively high production cost [25]. The microbial production of PHAs is also limited by batch variability, inadequate material properties, and challenges in controlling copolymer sequence [25].

Therefore, expanding the range of products that can be produced through microbial upcycling and aiming to create well-defined bespoke biopolymer constructs are highly desirable goals. For this purpose, *Pseudomonas* bacteria are particularly interesting, as they can robustly utilize single alkanes and alkane mixtures as a sole carbon source during growth [22, 26–28]. Moreover, synthetic biology tools exist for engineering *Pseudomonas* bacteria, including plasmid vectors and genomic integration methods [29, 30]. With regards to high value recombinant biopolymer products, silk proteins are amongst the most desirable biomaterials to target due to their unmatched combination of properties, including high toughness, strength, extensibility, biocompatibility, biodegradability, self-assembly capacity, thermal stability, solvent resistance, and ability to act as an optical waveguide [31–36]. Silk can rival or exceed the properties of conventional fossil-based plastics and can be manufactured into a diverse range of constructs, such as coatings, fibers, and hydrogels, facilitating their use in a wide variety of healthcare, food packaging, and sustainability applications.

In this work, we report the first microbial platform for converting polyethylene-derived hydrocarbons into bespoke recombinant protein products. We characterized and optimized the growth of *Pseudomonas* bacteria when hexadecane or chemically depolymerized polyethylene is used as the sole carbon nutrient source. We also created *Pseudomonas* strains with genomically integrated recombinant genes for green fluorescent protein (GFPuv) and a protein inspired by spider dragline spidroin. We show the ability of these strains to produce recombinant protein product in rich LB media as well as in minimal media supplemented with a model alkane, hexadecane, as the sole carbon source. Lastly, we perform proof-of-concept experiments demonstrating the ability of engineered *Pseudomonas* to effectively convert depolymerized polyethylene into GFPuv and dragline silk. Collectively, the findings reported herein establish a preliminary, yet functional, platform for the future upcycling of plastic waste to value-added recombinant proteins.

## Results and discussion

### Media design and growth of *Pseudomonas* strains using hexadecane as sole carbon source

Two strains of *Pseudomonas, P. aeruginosa* RR1 and *P. oleovorans,* were chosen for this work based on their previously demonstrated ability to utilize pyrolyzed polyethylene as a sole carbon source during growth (all strains, primers, and plasmids used in this work can be found in Additional file 1: Table S1) [22]. The growth of these strains was first characterized by using a model alkane, hexadecane (C16), as the sole carbon source. Hexadecane served as a proxy for hydrocarbons derived from polyethylene, as it can be a major alkane constituent resulting from the depolymerization of polyethylene via pyrolysis [22]. A minimal salt media that contained either ammonium chloride (NH_4_Cl) or ammonium nitrate (NH_4_NO_3_) as the nitrogen source was used, and hexadecane added was the sole source of elemental carbon in the media. The amount of hexadecane and elemental nitrogen supplied was based on theoretical calculations for the minimum amount of elemental carbon and nitrogen required to reach high cell densities of at least 10 g/L of wet pellet mass [37, 38]. Calculations were performed by assuming that dry cell mass accounted for 25% of wet pellet mass, and within that dry mass 50% was elemental carbon and 14% was elemental nitrogen [37, 38]. Thus, the theoretical minimum amount of hexadecane needed to supply enough elemental carbon to reach a wet pellet mass of 10 g/L is 1.47 g/L (0.147% w/v). Likewise, the theoretical minimum amount of ammonium chloride or ammonium nitrate required for a desired wet pellet mass of at least 10 g/L, is 1.34 and 1.00 g/L respectively.

A total of twelve different combinations of culture conditions were tested with three different amounts of hexadecane (0.46%, 4.6%, or 9.2% w/v), two different amounts of elemental nitrogen (0.65 g/L or 1.3 g/L elemental N), and two different nitrogen sources (NH_4_Cl or NH_4_NO_3_). Table 1 depicts the combinations of culture conditions that were tested for growth. To promote cell growth and recombinant protein production, the levels of supplied carbon and nitrogen were several times above the calculated minimum requirements for achieving a wet pellet mass of 10 g/L. Flask cultures were inoculated with a single plated colony of either *P. aeruginosa* RR1 or *P. oleovorans* and kept at 37 °C with 225 rpm shaking. As others have noted, the growth of *Pseudomonas* on hexadecane resulted in the formation of millimeter-sized white, waxy particulates that were numerous enough to interfere with OD600 measurements [39]. Thus, cell growth was quantified by performing serial dilutions of cultures with subsequent plating on LB agar to determine the density of colony-forming-units (cfu/ml).

**Table 1:** Design of Media for Growth on Hexadecane as Sole Carbon Source*

Hexadecane	Ammonium Chloride, NH_4_Cl	Ammonium Nitrate, NH_4_NO_3_
0.46% w/v or 4.6% w/v or 9.2% w/v	2.5 g/L (0.65 g/L elemental N) or 5 g/L (1.3 g/L elemental N)	1.9 g/L (0.65 g/L elemental N) or 3.8 g/L (1.3 g/L elemental N)

^*^The minimal salt media background maintains a constant composition for all conditions

**Fig. 1:**
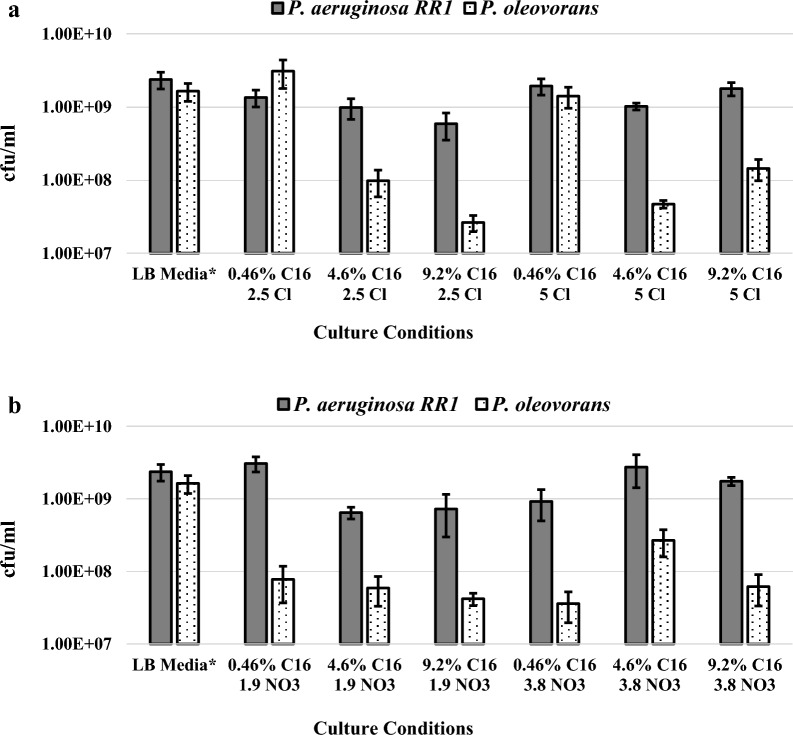
Average cfu/ml of *P. aeruginosa* RR1 and *P. oleovorans* 96 h after inoculation when using hexadecane as the sole carbon source (*18 h for LB media). Hexadecane (C16) was supplemented in the cultures at either 0.46%, 4.6%, or 9.2% (w/v). **a** Cfu/ml when 2.5 or 5 g/L of NH_4_Cl was supplied as the nitrogen source (2.5 Cl and 5 Cl). **b** Cfu/ml when 1.9 or 3.8 g/L of NH_4_NO_3_ was supplied as the nitrogen source (1.9 NO3 and 3.8 NO3). Error bars represent standard deviations from the mean values of three replicates

**Fig. 2:**
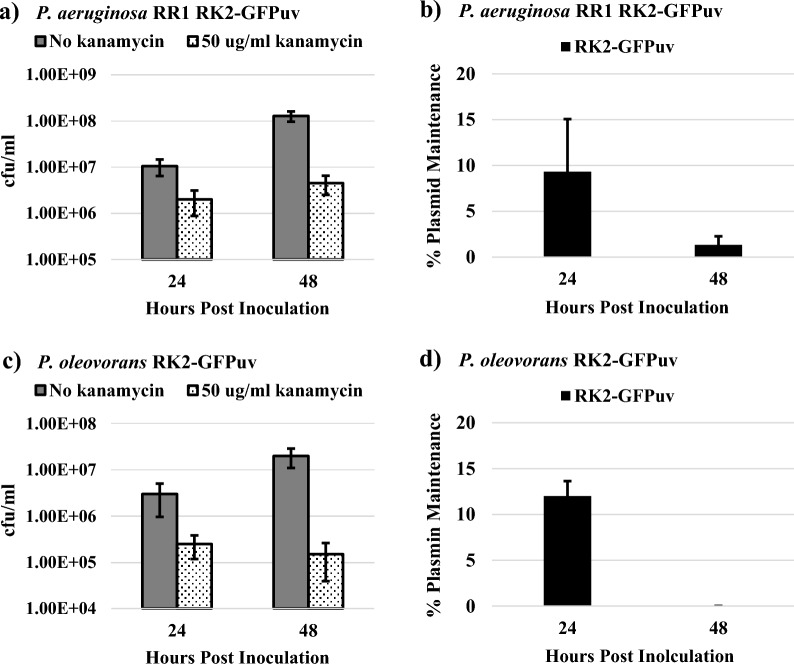
**a** Growth of *P. aeruginosa* RR1 RK2-GFPuv at 24 and 48 h post inoculation with and without kanamycin supplementation. **b** *P. aeruginosa* RR1 RK2-GFPuv plasmid maintenance at 24 and 48 h post inoculation in the presence of 50 µg/ml kanamycin. **c** Growth of *P. oleovorans* RK2-GFPuv at 24 and 48 h post inoculation with and without kanamycin supplementation. **d** *P. oleovorans* RK2-GFPuv plasmid maintenance at 24 and 48 h post inoculation in the presence of 50 µg/ml kanamycin. For both strains, culture conditions that yielded the best growth on hexadecane were used. Namely 0.46% w/v hexadecane with 1.9 g/L NH_4_NO_3_ for *P. aeruginosa* RR1 and 0.46% w/v hexadecane with 2.5 g/L NH_4_Cl for *P. oleovorans*. Error bars represent standard deviations from the mean values of three replicates

Figure 1 depicts the average cfu/ml of *P. aeruginosa* RR1 and *P. oleovorans* cultures 96 h after inoculation when using hexadecane as the sole carbon source under different culture conditions. For comparison, data for overnight cultures (18 h) grown in rich LB media can be found on the far left, averaging 2.38 ± 0.61 × 10^9^ cfu/ml for *P. aeruginosa* RR1 and 1.65 ± 0.45 × 10^9^ cfu/ml for *P. oleovorans*. This corresponds to OD600 values in LB media of 2.62 ± 0.19 for *P. aeruginosa* RR1 and 2.2 ± 0.16 for *P. oleovorans*. Seven out of the twelve different culture conditions facilitated the growth of *P. aeruginosa* RR1 to levels above 1 × 10^9^ cfu/ml, which is comparable to the dense growth observed for overnight cultures in LB media (Fig. 1). The five remaining conditions facilitate growth of to at least 5.9 × 10^8^ cfu/ml (Fig. 1). *P. oleovorans* was more limited in the number of conditions that facilitated robust growth, as only two conditions supported growth above 1 × 10^9^ cfu/ml and eight conditions showed cfu/ml below 1 × 10^8^ (Fig. 1). The two best conditions both contained the lowest concentration of hexadecane tested (0.46% w/v) and NH_4_Cl as the nitrogen source (Fig. 1). *P. oleovorans* showed a preference for NH_4_Cl versus NH_4_NO_3_, as three conditions using NH_4_Cl supported growth above 1 × 10^8^ cfu/ml but only one condition yielded such results when NH_4_NO_3_ was supplied (Fig. 2). With substantially higher cfu/ml across all but two culture conditions, *P. aeruginosa* RR1 was found to grow robustly on a wider range of culture conditions versus *P. oleovorans* (Fig. 1).

Growth kinetics for the two strains can be found in Additional file 1: Figures S2 and S3. For both *Pseudomonas* strains, the cfu/ml in cultures directly after inoculation with a plated colony was in the 10^5^ range. At 24 h post inoculation, cfu/ml of *P. aeruginosa* RR1 was found to be in the 10^7^ range, with an increase to the lower end of the 10^8^ cfu/ml range by 48 h (Additional file 1: Fig. S2). Measurements at 72 h showed growth increases to the high end of the 10^8^ cfu/ml range (Additional file 1: Fig. S2). At 96 h the cfu/ml in *P. aeruginosa* RR1 cultures was either maintained in the 10^8^ cfu/ml range or had increased to above 1 × 10^9^ cfu/ml (Figs. 1 and Additional file 1: Figure S2). The shape of the growth curves indicates that cells had reached either a late log phase or early stationary phase at 96 h (Additional file 1: Fig. S2). The growth kinetics of *P. oleovorans* showed more variability across culture conditions and required more time to reach the 10^7^–10^8^ cfu/ml range, versus *P. aeruginosa* RR1 (Figs. 1 and Additional file 1: Figure S3). At 24 h, *P. oleovorans* cfu/ml was observed to be in the low 10^6^ range, with either no substantial increase or an increase to the high 10^6^ range at 48 h (Additional file 1: Fig. S3). At 72 h, growth ranged from 6 × 10^6^ to 7.4 × 10^8^ cfu/ml depending on culture conditions (Additional file 1: Fig. S3). Likewise, at 96 h cultures ranged from 2.63 × 10^7^ to 3.1 × 10^9^ cfu/ml (Figs. 1 and Additional file 1: Figure S3). The shapes of the *P. oleovorans* growth curves indicate that the cultures may have been in a late log phase and shown additional growth over a longer time frame (Additional file 1: Fig. S3). Notwithstanding, this data shows that *P. aeruginosa* RR1 has more favorable growth kinetics (higher cfu/ml at 24 and 48 h) across the tested culture conditions versus *P. oleovorans*. These findings, combined with 1–2 order of magnitude differences in the final cfu/ml for the two species, lead to the conclusion that culture conditions must be specifically designed for a given species when an alkane is employed as the sole carbon source and growth trends observed in one species are likely not replicable in another.

### Deleterious effects of antibiotic selection and plasmid loss in hexadecane media

Upon identifying conditions that yielded growth of *P. aeruginosa* RR1 and *P. oleovorans* above 1 × 10^9^ cfu/ml with hexadecane as the carbon source, focus was turned to recombinant protein production. Green fluorescent protein (GFPuv) was chosen as a reporter protein to screen for proof-of-concept results before transiting to a recombinant silk construct. A plasmid-based approach was first tested, using vector pBb(RK2)1 k-GFPuv (RK2-GFPuv) [29]. The RK2-GFPuv vector was developed for use in *Pseudomonas* and contains an IPTG-inducible genetic circuit to produce a GFP mutant (GFPuv or eGFP) that has increased fluorescence over wild-type GFP for easier detection and quantification [29, 40]. The vector also contains a kanamycin resistance gene for selection [29]. The RK2-GFPuv plasmid was transformed into the *Pseudomonas* strains to create strains *P. aeruginosa* RR1 RK2-GFPuv and *P oleovorans* RK2-GFPuv. Production of GFPuv was first screened in rich LB media, with *P. aeruginosa* RR1 RK2-GFPuv and *P. oleovorans* RK2-GFPuv producing the recombinant protein at titers of 10.9 ± 2.7 and 12.4 ± 2.3 mg/L, respectively (Additional file 1: Fig. S4). From here, culture conditions that yielded the highest average cfu/ml at 96 h for strains grown on hexadecane were chosen for the plasmid-based conversion of hexadecane to a recombinant protein, namely, 0.46% w/v hexadecane with 1.9 g/L NH_4_NO_3_ for *P. aeruginosa* RR1 and 0.46% w/v hexadecane with 2.5 g/L NH_4_Cl for *P. oleovorans*.

However, upon addition of the antibiotic selection agent (kanamycin) to the hexadecane-based media, a severe inhibition of the growth of strains *P. aeruginosa* RR1 RK2-GFPuv and *P oleovorans* RK2-GFPuv was observed (Fig. 2a and c). At 24 h culture time, both *P. aeruginosa* RR1 RK2-GFPuv and *P. oleovorans* RK2-GFPuv showed approximately an order of magnitude decrease in cfu/ml when kanamycin was included in the media. By 48 h, there was a decrease in cfu/ml of approximately two orders of magnitude. Further examination showed that despite the presence of selective kanamycin, plasmid maintenance levels were found to be low in the system (Fig. 2b and d). Plasmid maintenance 24 h post inoculation was 9.3 ± 5.7% for *P. aeruginosa* RR1 RK2-GFPuv and decreased to 1.3 ± 0.9% by 48 h (Fig. 2b). Plasmid maintenance at 24 h post inoculation for *P. oleovorans* RK2-GFPuv was 12.0 ± 1.6% and decreased to 0.0 ± 0% by 48 h (Fig. 2d). This contrasts with cultures grown overnight (18 h) in LB media with kanamycin, which show plasmid maintenance levels of 79.3 ± 16.4% and 97.3 ± 0.9% for *P. aeruginosa* RR1 RK2-GFPuv and *P. oleovorans* RK2-GFPuv, respectively. The culture conditions for both strains were changed to 0.46% w/v hexadecane with 5 g/L NH_4_Cl (a condition that supported robust growth for both strains) to ascertain if the inhibited growth and plasmid maintenance could be alleviated by altering media composition. However, this made no substantial difference for growth or plasmid maintenance at 24 or 48 h post inoculation (Additional file 1: Figure S5).

It was hypothesized that the low copy number (3) of the RK2-GFPuv vector may cause difficulties with passage of the plasmid to daughter cells during growth in the hexadecane media [41]. Two new vectors, pBb(B5)1 k-GFPuv (B5-GFPuv) and pBb(RSF1010)1 k-GFPuv (RSF-GFPuv), were tested in *P. aeruginosa* RR1 (transformation of these vectors into *P. oleovorans* was unsuccessful) [29]. Plasmid B5-GFPuv has a copy number of 70, and RSF-GFPuv has a copy number of 30–60 in *Pseudomonas,* with both vectors containing an IPTG-inducible GPFuv gene and a kanamycin resistance gene [29, 42]. These increased copy numbers did not yield improved outcomes, as both vectors still showed only single digit percentages of plasmid maintenance 48 h post inoculation and there was no substantial improvement in culture cfu/ml at when selective kanamycin was present (Additional file 1: Fig. S6). Thus, it appears that the use of plasmids and antibiotic selection in *Pseudomonas* is not a feasible option for producing recombinant proteins in cultures with an alkane as a sole carbon source, independent of the culture conditions, specific species, or plasmid backbone.

### Genomic integration of recombinant GFPuv gene

A strategy that excluded antibiotic selection and enabled higher maintenance of recombinant genes over a 96-h period was required to achieve measurable levels of recombinant protein production when *Pseudomonas* was grown on hexadecane. Genomic integration was identified as a method that would meet these criteria, and an IPTG-inducible recombinant GFPuv gene with a tac promoter was genomically integrated into *P. aeruginosa* RR1 to create the strain *P. aeruginosa* RR1 g-GFPuv. Integration of the GFPuv construct was confirmed with colony PCR (Additional file 1: Fig. S7). The integration method utilizes two suicide vectors for the integration of constructs at a single *att*Tn*7* site within the *Pseudomonas* genome [30]. Mutants are isolated from wild type cells via the co-integration of a gentamycin resistance gene and subsequent plating on LB-gentamicin plates [30]. However, gentamicin is not required to maintain the genomic integration, which is reported to be stable for at least 100 generations [30]. Despite the successful transformation of the RK2-GFPuv vector into *P. oleovorans*, a genomic mutant of this species was unable to be created as neither transformation nor conjugation techniques could introduce the genomic integration suicide vectors into *P. oleovorans*. Thus, all subsequent work was performed with genomic mutants of *P. aeruginosa* RR1, however, future work should investigate new genomic integration techniques to analyze the performance of a genomically altered *P. oleovorans* strain. Experiments were first performed with rich LB media to ensure that recombinant expression of the integrated GFPuv gene could be achieved without gentamicin selection. Single colonies of *P. aeruginosa* RR1 g-GFPuv taken from LB-gentamicin plates were used to inoculate flask cultures of LB media (without gentamicin) that contained either 0, 0.3, or 1 mM IPTG. After overnight incubation (18 h) at 37 °C cells were harvested and the presence of fluorescent GFPuv can be seen when the lysates are exposed to UV light (Additional file 1: Figure S8). Titers of GFPuv expressed from the genome were found to be 9.0 ± 1.6 mg/L with 0.3 mM IPTG and 9.9 ± 1.1 mg/L with 1 mM IPTG, which is similar to the GFPuv titer of 10.9 ± 2.73 mg/L when using the low-copy number RK2-GFPuv plasmid.

### Conversion of hexadecane to recombinant GFPuv and spider silk protein

Although certain hexadecane culture conditions yielded more growth versus others for strain *P. aeruginosa* RR1, all tested conditions facilitated growth beyond 5 × 10^8^ cfu/ml by 96 h and it was unknown if the best growth conditions would also result in the highest yields of recombinant protein. Likewise, the optimal time at which to induce cells with IPTG was unknown. Therefore, a fluorescence assay was developed to measure GFPuv titer directly from unpurified cell lysates to enable the testing of many conditions. Using 96 h total culture times, all twelve culture conditions that were tested for growth were also tested for recombinant GFPuv production. The twelve different culture conditions were each tested using three different expression conditions. They were induced with 0.3 mM IPTG at either 24-, 48-, or 72-h culture time (corresponding to 72-, 48-, and 24- hour expression times), thus, resulting in data for 36 different conditions for the conversion of hexadecane to recombinant GFPuv.

**Fig. 3:**
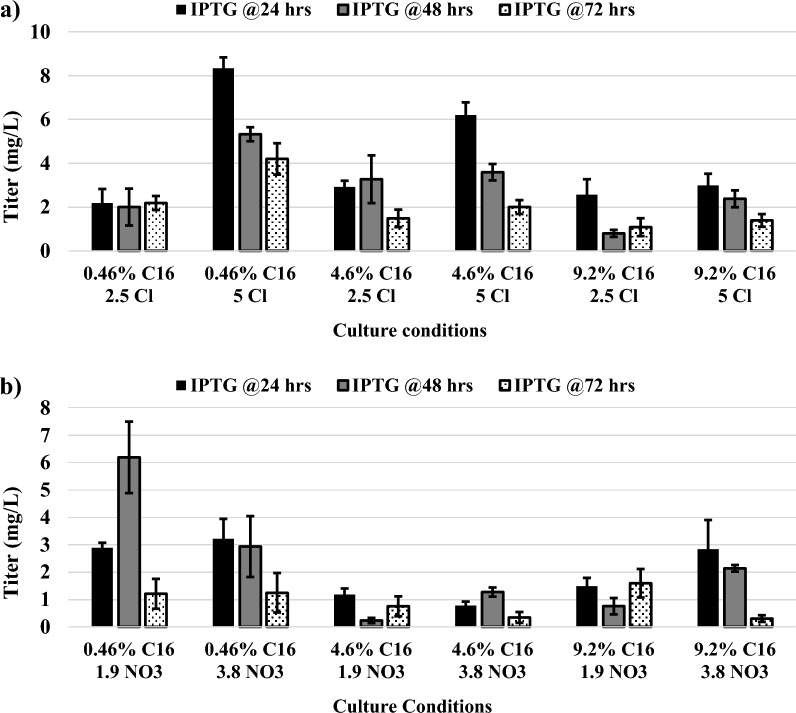
Titers of recombinant GFPuv when strain P. aeruginosa RR1 g-GFPuv is grown on hexadecane under a variety of culture conditions. **a** Cultures grown with either 2.5 or 5 g/L of NH_4_Cl (2.5 Cl and 5 Cl). **b** Cultures grown with either 1.9 or 3.8 g/L of NH_4_NO_3_ (1.9 NO_3_ and 3.8 NO_3_). Error bars represent standard deviations from the mean value of three replicates. 0.3 mM IPTG was added at 24, 48, or 72 h post inoculation. Total culturing time was 96 h, resulting in expression times of either 72, 48, or 24 h. Hexadecane concentrations (C16) are provided as % w/v

Production of GFPuv from hexadecane can be seen in Additional file 1: Figure S9, in which lysates of induced *P. aeruginosa* RR1 g-GFPuv show a green fluorescence under UV light as compared to pale blue fluorescence of uninduced cultures (the pale blue color is due to production of a natural pigment, pyocyanin) [43]. Figure 3 shows the GFPuv titer across all culture and expression conditions tested. The highest titer of 8.3 ± 0.5 mg/L was identified in culture conditions of 0.46% w/v hexadecane with 5 g/L NH_4_Cl that had been exposed to expression conditions of 0.3 mM IPTG added 24 h post inoculation (72-h expression time). This is approximately 92% of the GFPuv production level observed for *P. aeruginosa* RR1 g-GFPuv when grown in LB media with 0.3 mM IPTG. The second highest titers achieved for the conversion of hexadecane to recombinant GFPuv were approximately 25% less than this (Fig. 3). Therefore, 0.46% w/v hexadecane with 5 g/L NH_4_Cl was selected as the optimal culture condition for the conversion of hexadecane to a recombinant silk protein. Figures 3 and Additional file 1: Fig. S9 also show that the time of induction affected GFPuv titer, with substantial decreases in visible fluorescence for titers below 4 mg/L. IPTG added at 24 or 48 h post inoculation (longer expression times) yielded increased titers versus IPTG added at 72 h for ten of the twelve different media compositions (Fig. 3). Longer expression times may be favorable for titers due to the slow growth kinetics of this system in comparison to cultures grown in rich media (Fig. 1). Results also show that when comparing cultures with identical hexadecane concentration, elemental nitrogen concentration, and IPTG induction times, those containing NH_4_Cl achieved higher GFPuv titers versus those containing NH_4_NO_3_ in 15 out of 18 cases (Fig. 3). This finding is significant when considering that there is no significant difference in growth of *P. aeruginosa* RR1 on hexadecane when either NH_4_Cl or NH_4_NO_3_ is used (Figs. 1 and Additional file 1: Fig. S2). Counterintuitively, the single highest GFPuv levels achieved for both nitrogen sources used the lowest concentration of C16 (0.46% w/v) (Fig. 3). It is known that PHA synthesis in *Pseudomonas* is promoted by increasing the ratio of carbon to nitrogen in media, and this may shuttle cellular resources away from non-essential recombinant protein synthesis thus lowering titers [44, 45]. Future work using ^13^C-labeled hexadecane to do a metabolic-flux analysis may resolve differences in metabolism at various hexadecane concentrations and lead to a more developed understanding of this observation [46].

We have previously produced the de novo designed recombinant silk protein, A5 4mer, in *E. coli* and this construct was genomically integrated into *P. aeruginosa* RR1 to form *P. aeruginosa* RR1 g-A5 [47]. The A5 4mer primary sequence (Additional file 1: Fig. S1) was designed to represent a truncated version of dragline silk protein produced by spiders [47]. Expressions carried out in LB media demonstrated that *P. aeruginosa* RR1 g-A5 was able to produce the A5 4mer protein at a titer of 14.7 ± 1.57 mg/L when overnight cultures (18 h) inoculated from a single colony were grown at 37 ℃ in the presence of 1 mM IPTG. This also represented the first production of recombinant silk in the *Pseudomonas* genus. Unlike GFPuv, for which small amounts could be measured with fluorescence, a larger amount of A5 4mer protein was required for accurate measurements of titer. Therefore, larger culture volumes were employed for the conversion of hexadecane to recombinant silk. A single colony of *P. aeruginosa* RR1 g-A5 was used to inoculate a 25 ml starter culture of 0.46% w/v hexadecane with 5 g/L NH_4_Cl. After 72 h of culture time, the entire 25 ml starter culture was used to inoculate a 200 ml expression culture containing 0.46% w/v hexadecane with 5 g/L NH_4_Cl and supplemented with either 0.3 or 1 mM IPTG. The expression culture was then incubated for 48, 60, or 72 h.

**Fig. 4:**
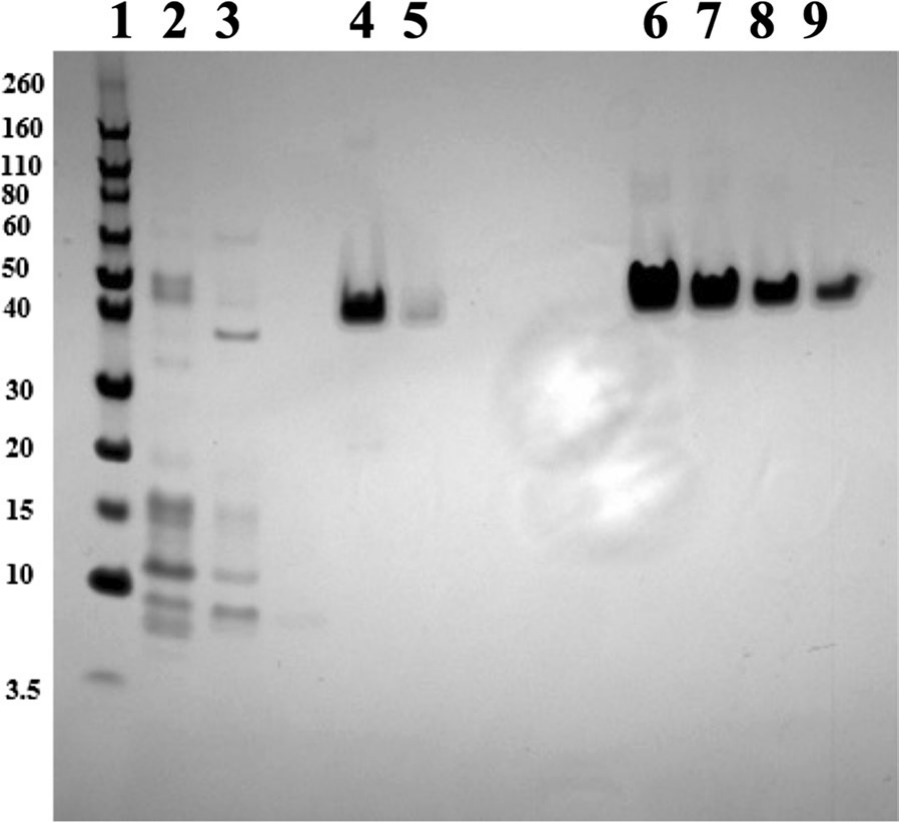
SDS PAGE after nickel chromatography showing the A5 4mer silk protein purified from lysates of *P. aeruginosa* RR1 g-A5 that were grown using hexadecane as the sole carbon source (culture conditions of 0.46% w/v hexadecane with 5 g/L NH_4_Cl and induced with 1 mM IPTG). Expression time was 60 h. Lanes (1) Protein ladder, with mass listed to left in kDa (2) flow through (3) wash (4) first elution fraction (5) second elution fraction (6–9) standards of A5 4mer protein, 2, 1, 0.5, and 0.25 mg/L. The band in lane 4 (first elution fraction) is shown at an identical molecular weight (~ 38 kDa) as the standards of A5 4mer protein produced in *E. coli* (lanes 6–9). As previously documented, the 16 kDa A5 4mer silk protein appears at ~ 38 kDa due to its high level of structural disorder and subsequent aberrant mobility through SDS PAGE [47]. A slight smiling effect was observed on the SDS PAGE, resulting in a slightly slower migration of outer lanes (Lanes 1,2 and 5–8) compared to inner lanes (Lane 4) [48, 49]. The faint protein band to the right of Lane 4 is the remainder of histidine-tagged silk protein that was still bound to the purification resin after the Lane 4 elution

**Fig. 5:**
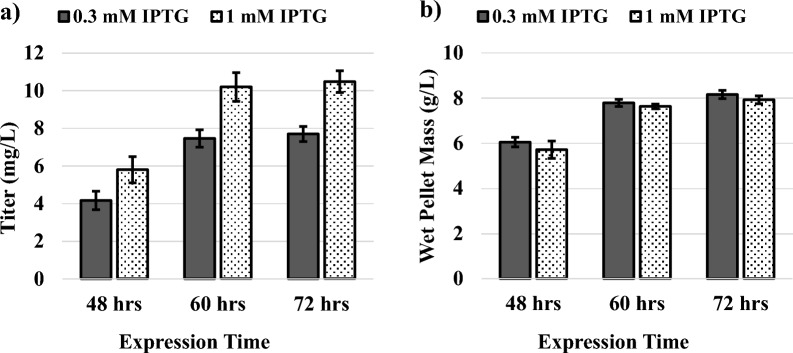
**a** Titer of the A5 4mer produced in *P. aeruginosa* RR1 g-A5 grown on hexadecane (culture conditions of 0.46% w/v hexadecane with 5 g/L NH_4_Cl). Expression times were either 48, 60, or 72 h and cultures were induced with either 0.3 or 1 mM IPTG. **b** Wet pellet mass at the end of A5 4mer expressions. Error bars represent standard deviations from the mean value of three replicates

Figure 4 shows A5 4mer silk protein purified from a culture of *P. aeruginosa* RR1 g-A5 grown using hexadecane as the sole carbon source and an expression time of 60 h. The purified protein can be seen at an identical molecular weight as standards of the protein produced in *E. coli* (Fig. 4). As previously documented, the 16 kDa A5 4mer silk protein appears at ~ 38 kDa due to its high level of structural disorder and subsequent aberrant mobility through SDS PAGE [47]. This represents the first conversion of an alkane to a recombinant silk protein or protein-based material. The highest titers of approximately 10 mg/L were achieved at 60- or 72-h expression time and 1 mM IPTG, with only a negligible difference between two expression times (Fig. 5). Interestingly, the similarity in titer between the 60- and 72-h mark coincided with a plateau of the wet pellet mass at harvest. The change in wet pellet mass from 48 to 60 h was + 1.9 g/L, but only + 0.3 g/L from 60 to 72 h (Fig. 5). Increasing the IPTG concentration from 0.3 mM to 1 mM increased the silk protein titer by approximately 41% and 36% for the 48- and 60/72-h expressions, respectively. The expression cultures were observed to be relatively clear directly after inoculation with the starter culture. However, progressive increases in turbidity were observed over time in accordance with increases in cfu/ml, with cultures becoming completely opaque at the time of harvest (Additional file 1: Fig. S10). Expression cultures developed pale orange color that progressed to a darker, burnt orange tone over time (Fig. S10).

### Conversion of polyethylene-derived hydrocarbons to recombinant silk protein

Samples of depolymerized polyethylene were obtained to determine if our results could be extended to plastic-derived substrates. Polyethylene (*M*_w_ = 4,000 g/mol, *M*_n_ = 1,700 g/mol, and *Đ* = 2.35) was depolymerized via catalytic hydrogenolysis using a Pt/SrTiO_3_ catalyst in a batch reactor at 300 °C, in presence of 170 psi of H_2_ for 72 h under solvent-free conditions [50, 51]. This method confers control over the size and dispersity of the products and was leveraged to produce hydrocarbons within the range that *P. aeruginosa* can metabolize (approximately C_8_-C_27_) [22, 50, 51]. This is the first report to use such a method for the generation of a microbial feedstock from a plastic substrate. Previous reports have used methods such as pyrolysis or alkaline hydrolysis [14, 22]. It should be noted that unlike the monodisperse and linear hexadecane used previously herein, the chemically depolymerized polyethylene sample created in this study contains a distribution of alkanes (*M*_w_ = 305 g/mol, *M*_n_ = 240 g/mol, and *Đ* = 1.26) and a non-negligible degree of branching (N_branch/1000C_ = 111). The sample is white and has the consistency of a soft wax (Additional file 1: Fig. S11). Several experiments were performed to establish robust growth of *P. aeruginosa* RR1 g-A5 when using this sample as the sole carbon source. For all growth experiments, the supplemental nitrogen was held constant at 5 g/L NH_4_Cl, as this provided the best GFPuv titers and facilitated the conversion of hexadecane to recombinant silk.

**Fig. 6:**
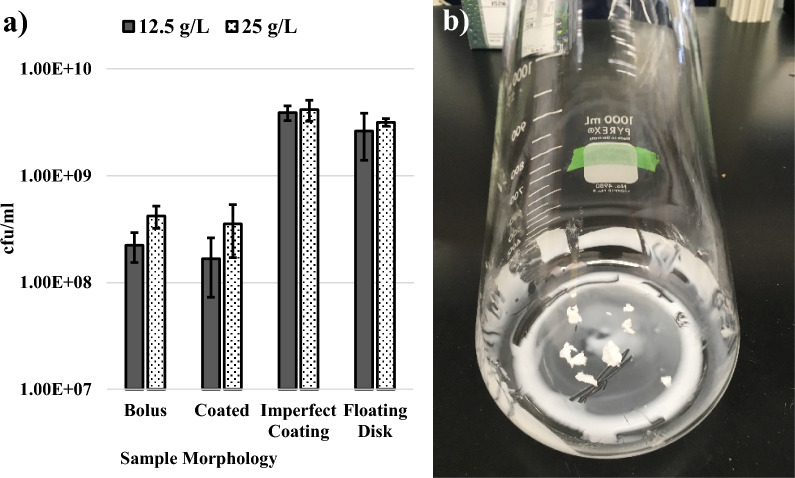
**a** Average cfu/ml 96 h post inoculation for *P. aeruginosa* RR1 g-A5 grown using depolymerized polyethylene as the sole carbon source. Multiple concentrations (12.5 and 25 g/L) and morphologies of the depolymerized polyethylene were tested, with 5 g/L NH_4_Cl supplied as the nitrogen source. Error bars represent standard deviations from the mean values of three replicates. **b** Depiction of the “Imperfect Coating” preparation method. Samples of the depolymerization polyethylene were added to flasks and gently melted to form a coating on the bottom. Upon solidification, the coatings were agitated to create an imperfect coating coverage. Sections of coating that were removed were added back into the flask as small boluses to maintain consistency in the total amount of sample per liter of culture

Figure 6a depicts the average cfu/ml 96 h after inoculation for *P. aeruginosa* RR1 g-A5 grown using the depolymerized polyethylene sample as the sole carbon source supplied at either 12.5 or 25 g/L. Preliminary experiments were performed by simply depositing centimeter-scale chunks of the depolymerized polyethylene sample into culture flasks, with cohesion of the sample resulting in a floating bolus (Fig. 6a “bolus”, Fig S12a). Growth of *P. aeruginosa* RR1 g-A5 using this method was suboptimal compared to results obtained with hexadecane, reaching cfu/ml levels several times below that of optimized conditions using hexadecane (Figs. 1 and 6a). It was hypothesized that increasing the surface area of the depolymerized polyethylene sample may allow the cells improved access to the substrate and facilitate increased growth. As such, multiple alternative morphologies of the depolymerized polyethylene sample within the culture flask were tested (Additional file 1: Fig. S12). A hot plate was used to melt the sample and coat the bottom of the culture flasks prior to the addition of media and cells. This resulted in coatings that contained no gaps in the coverage of the flask within their area (Additional file 1: Fig. S12b). Although the sample surface area was increased relative to a bolus, no improvements in growth over 96 h were observed (Fig. 6a “coated”). However, growth was substantially improved if the coating was manually disrupted with a sterile metal laboratory scoop to form an imperfect coating with gaps in the coverage of the flask (Fig. 6a “imperfect coating”, Fig. 6b, Additional file 1: Fig. S12d). This method yielded an average cfu/ml of 3.9 ± 0.6 and 4.2 ± 0.9 × 10^9^ at 96 h post inoculation for 12.5 and 25 g/L of substrate, respectively. While functional, it should be acknowledged that the preparation of the “imperfect coating” via manual disruption with a laboratory scoop was not standardized and was thus subject to variability in terms of final morphology. Future work should seek to prepare hydrocarbon substrates into well-defined geometries with high surface area, such as micro or nano-scale particles. Notwithstanding, this imperfect coating method surpasses the highest levels of growth achieved with optimized conditions using hexadecane as the sole carbon source (Figs. 1 and 6a). To our knowledge, this is the highest reported level of cell growth achieved for a microbial strain grown on a polyethylene-derived, or alkane-based, substrate as the sole carbon source. A final sample preparation method was also tested, in which a bolus of the sample was melted on top of heated culture media placed in a flask (prior to inoculation). Upon cooling, the sample formed a solid, thin floating disk that could be broken into smaller pieces with a pipette tip or prolonged agitation in an incubator (Fig. 6a “floating disk”, Additional file 1: Fig. S12c). This method also yielded robust cfu/ml at 96 h, although slightly lower than an imperfect coating (Fig. 6a). Moreover, it was observed that pieces of the “floating disk” substrate tended to adhere to upper regions of the flask upon shaking incubation, effectively removing them from the culture media unless manually pushed down. For these reasons, the method of generating an imperfect coating of the substrate was chosen for silk protein expressions. Likewise, 12.5 g/L was chosen instead of 25 g/L due to similar growth between the two conditions and the ability to conserve the depolymerized polyethylene sample (Fig. 6a “imperfect coating”).

**Fig. 7:**
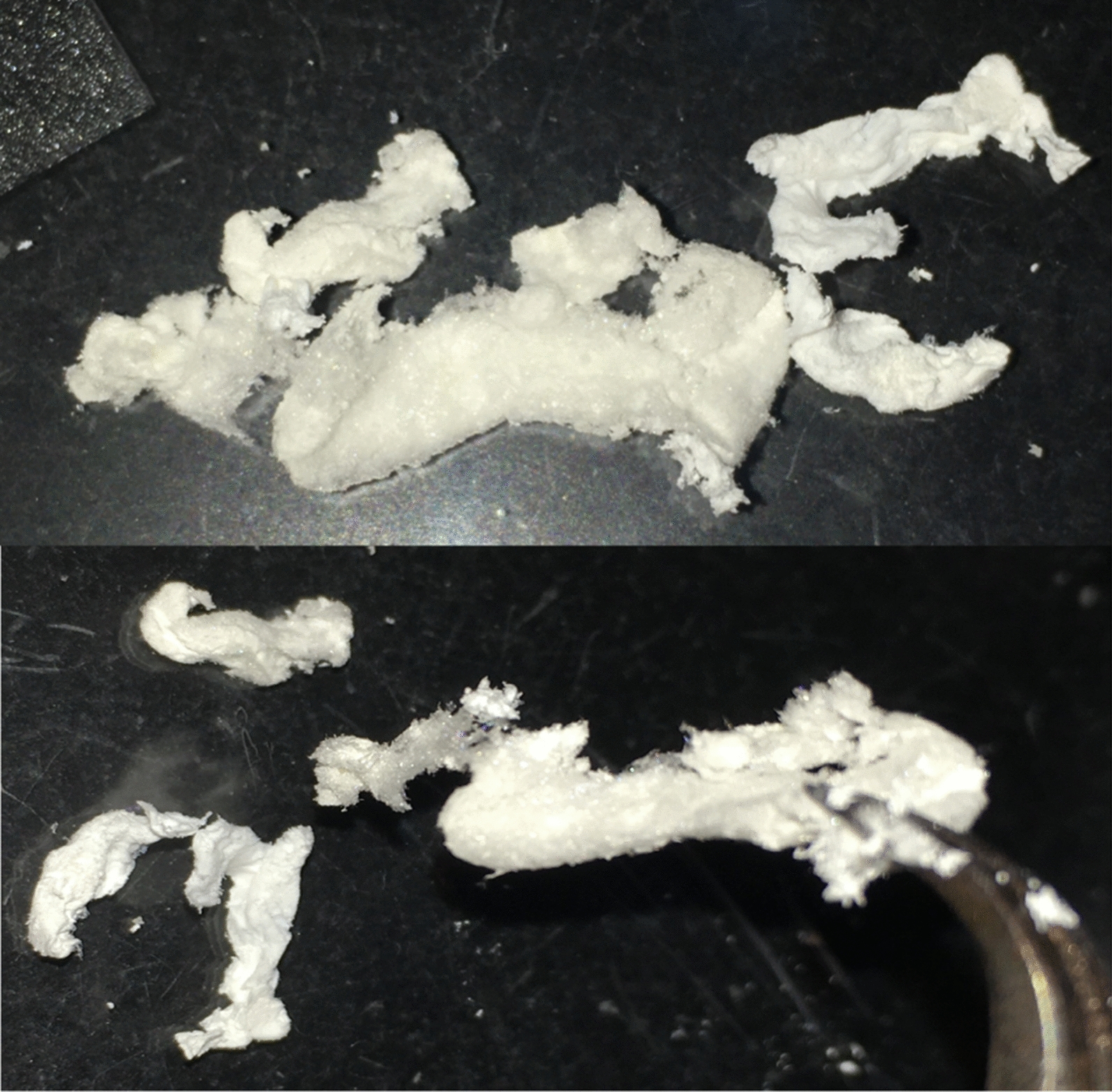
Purified A5 4mer spider silk protein produced through the microbial conversion of depolymerized polyethylene. The silk protein was dialyzed into water and lyophilized, resulting in several discrete and cohesive chunks of a white, polymeric material that ranged in length from approximately 1.5–4 cm. This image was obtained directly after the freeze-drying process, and the protein sample had not been spun into fibers or put through any additional material processing

An expression strategy derived from results using hexadecane was applied for the conversion of polyethylene to recombinant silk. A 25 ml starter culture was grown for 72 h, and the entire culture was used to inoculate 200 ml of fresh media (with 1 mM IPTG) for a 72-h expression period. Both the starter cultures and expression cultures contained 12.5 g/L of depolymerized polyethylene prepared using the imperfect coating method. Akin to expressions using hexadecane, cultures utilizing depolymerized polyethylene were clear upon inoculation and increased in turbidity over time in accordance with cell growth, resulting in extremely turbid cultures at harvest (Additional file 1: Fig. S13). At harvest, however, expression cultures utilizing depolymerized polyethylene were observed to be a pale brown color versus the burnt orange observed when hexadecane was used (Additional file 1: Fig. S13d). Nickel chromatography on lysates of expression cultures of *P. aeruginosa* RR1 g-A5 grown using depolymerized polyethylene as the sole carbon source showed the A5 4mer protein in the elution fraction, (Additional file 1: Fig. S14) thus demonstrating the conversion of polyethylene to a recombinant protein-based polymer material. Titers of the silk protein were 11.3 ± 1.1 mg/L, which is approximately 77% of the production level observed in rich LB media for strain *P. aeruginosa* RR1 g-A5. Samples of the A5 4mer spider silk protein produced via the microbial conversion of depolymerized polyethylene were dialyzed into water and lyophilized, resulting in white, cohesive biopolymer material (Fig. 7).

In conclusion, this work demonstrates the first reported microbial platform for the conversion of polyethylene-derived substrates to a bespoke recombinant protein. Our results also demonstrate the first recombinant silk production in the *Pseudomonas* genus. Additionally, culture conditions and specific polyethylene depolymerization parameters that yield levels of microbial growth using a plastic-derived substrate as the sole carbon source are identified. Although a plasmid-based expression system was inefficient, genomic integration of recombinant genes was found to be a viable strategy for facilitating the microbial conversion of depolymerized polyethylene into recombinant proteins. Taken together, these findings demonstrate the modularity this novel system, which can potentially be used to upcycled waste polyolefins into any recombinant protein that can be integrated into the genome of *P. aeruginosa* RR1.

Future work will seek to increase production levels of recombinant constructs through optimization of the recombinant gene. In this context, multi-copy integrations of heterologous genes may increase production levels compared to the single copy integrations reported in this work. Likewise, the use of a stronger promoter on recombinant integrations (such as T7) may yield increased titers compared to the tac promoter used in this work. Additionally, future work may seek to decrease the time required for *P. aeruginosa* RR1 to reach > 1 × 10^9^ cfu/ml when depolymerized polyethylene is the sole carbon source, thus increasing the efficiency and decreasing the cost of the process. Within this scope, fundamental work that can identify metabolic bottlenecks when *Pseudomonas* is grown on alkane-based substrates by way of metabolic flux analysis (using ^13^C-labeled feedstocks) and quantify the amount of substrate consumed by the cells and its conversion rate to recombinant protein are also important aspects of future work [46]. Potential strategies to increase the rate of growth include the testing of additional morphologies of the depolymerized polyethylene sample or upregulation of endogenous alkane hydroxylases within *Pseuodmonas* [28]. Furthermore, the use of a depolymerization process that yields oxidized hydrocarbon substrates of increased solubility may be advantageous by promoting faster uptake of the carbon source into the cells. Oxidized substrates can also bypass the first, and possibly rate-limiting, step of terminal oxidation that occurs in the alkane degradation pathway used by *Pseudomonas* [52, 53]*.* Since *P. aeruginosa* is an opportunistic pathogen, future work should also seek to implement non-pathogenic strains of *P. aeruginosa* to promote the optimization and scale-up of this process by ensuring a high degree of safety [54]. Recent work shas hown that the virulence of *P. aeruginosa* can be attenuated by deleting five chromosomal genes (*toxA*, *plcH*, *phzM*, *wapR*, and *aroA*) through a plasmid-based methods [55]. This resultant strain exhibits a mortality rate of 0% in mice upon injection, as compared to a 95% mortality rate of wild-type *P. aeruginosa* [55]*.* However, it is unknown if these genetic changes will impact the efficiency of cell growth and recombinant protein production when hydrocarbon-rich substrates are used as the sole carbon source.

## Materials and methods

### Strains, plasmids and primers

Descriptions and sources for all strains, plasmids, and primers (including primer sequences) used in this study can be found in Additional file 1: Table S1. Chemically competent *E. coli* DH5α was used for restriction cloning experiments and plasmid storage (Thermo Fisher Scientific, Waltham, MA). Restriction enzymes and T4 DNA ligase were purchased from Thermo Fisher Scientific (Waltham, MA) and DNA manipulations were carried out using the manufacturer’s protocols. Extraction of plasmid DNA from *E. coli* and from agarose gel was performed using the E.Z.N.A. Plasmid DNA Mini Kit I and the E.Z.N.A. Gel Extraction kit, respectively (Omega Bio-tek, Norcross, GA). Plasmids were introduced into the expression strains via electroporation using the Gene Pulser Xcell Electroporation System at (Bio-Rad, Hercules, CA). Both replicable and suicide plasmid vectors were introduced into *P. aeruginosa* RR1 and *P. oleovorans* using a standard electroporation protocol [56]. Transformants were isolated on LB agar plates supplemented with either 50 µg/ml kanamycin or 30 µg/ml gentamicin.

Genomic mutants of *P. aeruginosa* RR1 were created according to a previously published protocol [30]. For integration of the GFPuv gene, the primers GFPuv-Gf and GFPuv-Gr were used to PCR amplify the GFPuv gene from pBb(RK2)1 k-GFPuv. The amplicon was then cloned into pUC18-mini-Tn7T-LAC using the BcuI and HindIII restriction sites to create pUC18-Tn7T-GFPuv. Vectors pUC18-Tn7T-GFPuv and pTNS2 were then simultaneously transformed into *P. aeruginosa* RR1 to create strain *P. aeruginosa* RR1 g-GFPuv. Correct integration of the GFPuv gene was performed according to a previously published protocol and was done with colony PCR using the glmS-up and glmS-down primers [30]. For integration of the recombinant A5 4mer silk gene, primers A5-19bf and A5-19br were used to PRC amplify the A5 4mer gene from a pET-19b backbone [47]. The amplicon was then cloned into the pBb (RK2)1 k backbone (pBb(RK2)1 k-GFPuv with the GFPuv gene removed via digestion with BgIII and XhoI) using enzymes BgIII and XhoI to create pBb(RK2)1 k-A5. Primers A5-Gf and GFPuv-Gr were then used to PCR amplify the A5 4mer sequence from pBb(RK2)1 k-A5. The amplicon was subsequently cloned into pUC18-mini-Tn7T-LAC using the SacI and HindIII restriction sites to create pUC18-Tn7T-A5. Vectors pUC18-Tn7T-A5 and pTNS2 were then simultaneously transformed into *P. aeruginosa* RR1 to create strain *P. aeruginosa* RR1 g-A5.

### Growth. plasmid maintenance, and protein expressions

All cultures used in this study were incubated at 37 °C in Erlenmeyer flasks with 225 rpm shaking unless otherwise noted. LB media (Lennox broth, Sigma Aldrich, St. Louis, MO) was used for experiments in rich media. A minimal salt media background was used for all work performed when hexadecane or depolymerized polyethylene was supplied as the sole carbon source. The minimal salt media contained 9 g/L Na_2_HPO_4_⋅12H_2_O, 1.5 g/L KH_2_PO_4_, 0.2 g/L MgSO_4_⋅7H_2_O, 20 mg/L CaCl_2_, 4 mg/L ZnSO_4_⋅7H_2_O, 10 mg/L FeSO_4_⋅7H_2_O, 1 mg/L CuCl_2_⋅2H_2_O, 1 mg/L MnCl_2_⋅4H_2_O, 1 mg/L Na_2_B_4_O_7_⋅10H_2_O, 0.2 mg/L NiCl_2_⋅6H_2_O, 0.3 mg/L Na_2_MoO_4_⋅2H_2_O. Elemental nitrogen was supplied as either 2.5 or 5 g/L of NH_4_Cl, or either 1.9 or 3.8 g/L of NH_4_NO_3_. Hexadecane was supplied at either 0.46, 4.6, or 9.2% w/v. Depolymerized polyethylene was supplied at either 12.5 or 25 g/L. Hexadecane or depolymerized polyethylene was the only source of elemental carbon in the media. Data for cfu/ml over time was collected by first inoculating 25 ml cultures in 125 ml Erlenmeyer flasks with a single plated colony. Serial dilutions of cultures on the order of 10^3^–10^6^ and subsequent plating on LB agar plates were used to determine the cfu/ml in cultures. For growth studies using hexadecane or depolymerized polyethylene media, cfu/ml data points were taken a 0, 7, 24, 33, 48, 55, 72, 79, and 96 h post inoculation. For studies using rich LB media, cfu/ml measurements were taken at 18 h post inoculation. Plasmid maintenance was measured by patch plating 50 colonies from LB agar plates to LB agar plates supplemented with 50 µg/ml of kanamycin and incubating overnight at 37 °C. The percentage plasmid maintenance was calculated by counting the number of colonies that had grown on the selective plate and dividing by 50.

For plasmid-based expression of GFPuv in rich media, 2 ml of overnight cultures supplemented 50 µg/ml of kanamycin of *P. aeruginosa* RR1 RK2-GFPuv or *P. oleovorans* RK2-GFPuv were used to inoculated 50 ml expressions cultures. The 50 ml cultures were induced with 0.3 mM IPTG at an OD600 of 0.6 and moved to 20 ℃ for a 16-h expression. For expression of GFPuv or A5 4mer silk protein in rich media using *P. aeruginosa* RR1 g-GFPuv and *P. aeruginosa* RR1 g-A5, cultures supplemented with IPTG were inoculated with a single plated colony and cultured for 18 h. For expressions of GFPuv in hexadecane media, 25 ml cultures in 125 ml Erlenmeyer flasks were inoculated with a plated colony of *P. aeruginosa* RR1 g-GFPuv. 0.3 mM IPTG was added to the cultures at 24, 48, or 72 h post inoculation and total culture time was 96 h. For expression of A5 4mer silk protein in hexadecane media, a 25 ml preculture was inoculated from a single plated colony and incubated for 72 h. The entire preculture was used to inoculate 200 ml of fresh media supplemented with either 0.3 or 1 mM IPTG. The fresh culture was then cultured for 48, 60, or 72 h. For expression of A5 4mer silk protein in depolymerized polyethylene media, a 25 ml preculture was inoculated from a single plated colony and incubated for 72 h. The entire preculture was used to inoculate 200 ml of fresh media supplemented with 1 mM IPTG and subsequently cultured for 72 h.

### Lysis and titer quantification

For all GFPuv expressions, lysis was performed using the B-PER™ II Bacterial Protein Extraction Reagent (Thermo Fisher Scientific, Waltham, MA) according to the manufacturer’s protocol. GFPuv titer from expressions in rich media was calculated in an identical fashion to previously described methods [47]. Briefly, the size of the GFPuv band at 28 kDa on an SDS PAGE was analyzed in comparison to standards of bovine serum albumin using the ChemiDoc XRS + Image Lab v 6.0.1 software to determine titer (Bio-Rad, Hercules, California) [49]. From here, a fluorescence assay was used for measurements of GFPuv titer in hexadecane-based media. Serial dilutions using DI water (16x, 32x, 64x, 128x, 256x, 512x, 1024x, 2048x) were performed on both induced and uninduced lysates of *P. aeruginosa* RR1 g-GFPuv grown in rich media. The fluorescence of the samples was measured by inserting 200 µl aliquots of the dilutions into black, clear bottom 96 well plates and subsequent analysis with a PerkinEkmer EnVision 2104 Multilabel Reader and the Wallac Envision Manager v 1.12 software (Waltham, MA). Measurement parameters included a 405 nm excitation filter, a 535 nm emission filter, 5 mm measurement height, 75% excitation light, 75% second excitation light, 200 detector gain, 200 s detector gain, and 25 flashes. The change in fluorescence between uninduced and induced cultures was compared to the known GFPuv concentration to generate a standards curve that was used to calculated GFPuv titer from the fluorescence of culture lysates. Titers of GFPuv for cultures using hexadecane were calculated by diluting raw lysates 128 × in DI water and performing triplicate fluorescence measurements. For a given culture condition, the change in fluorescence between uninduced and induced cultures was used to derive GFPuv titer.

For expression of the 10 × histidine tagged A5 4mer silk protein, lysis and purification was performed using previously described methods [47]. Briefly, cells were centrifuged down before resuspension in a lysis buffer and frozen overnight. Upon thawing, cells were mixed with lysozyme and sonicated. The raw lysates were then exposed to a heat treatment at 80 °C before being centrifuged. Purification of the A5 4mer was conducted with 2 ml of HisPur Ni–NTA Resin (Thermo Fisher Scientific, Waltham, MA) that was placed in a 10 ml gravity-flow polypropylene chromatography column. Lysates were mixed with one part binding buffer (5 mM imidazole, 0.5 M NaCl, 20 mM Tris–HCl, pH 8) and passed through the column, washed with 6 ml of wash buffer (20 mM imidazole, 0.5 M NaCl, 20 mM Tris–HCl, pH 8). For experiments using hexadecane as the carbon source, silk protein was first collected by passing 3 ml of elution buffer (300 mM imidazole, 20 mM Tris–HCl, pH 8) through the column (first fraction). A subsequent elution (second fraction) was collected in a separate tube by passing an additional 3 ml of elution buffer through the column. For experiments using depolymerized polyethylene as the carbon source, silk protein was first collected by passing 6 ml of elution buffer (300 mM imidazole, 20 mM Tris–HCl, pH 8) through the column (first fraction). A subsequent elution (second fraction) was collected in a separate tube by passing an additional 6 ml of elution buffer through the column. Titer of the A5 4mer protein was found using the ChemiDoc XRS + Image Lab v 6.0.1 software and was performed in an identical fashion to previously described methods [47]. All SDS PAGEs for both GFPuv and A5 4mer were performed using previously described methods [47]. Purified A5 4mer protein was dialyzed into DI water using SnakeSkin™ 3.5 kDa Dialysis Tubing (Thermo Fisher Scientific, Waltham, MA) according to the manufacturer’s protocol. Samples were then frozen at -80 ℃ and then lyophilized.

### Production of the depolymerized polyethylene sample using pt/srtio_3_ catalyst catalyst synthesis

The procedure for the synthesis of the SrTiO_3_ (STO) catalyst support was performed according to previously described methods [50]. The STO pretreatment and catalyst synthesis were performed according to previously described methods [51]. The STO support was calcined at 550 °C for 4 h, treated with ozone in oxygen (8% O_3_) at 200 °C for 1.5 h, treated with steam at 200 °C for 2 h, and vacuum dried at 200 °C for 12 h. The STO nanocuboids were then suspended in a solution of platinum(II)acetylacetonate (Pt(acac)_2_) in toluene at 80 °C for 72 h under an inert atmosphere. The sample was washed with toluene and pentane, vacuum dried at 60 °C overnight, calcined at 300 °C in air for 4 h, and reduced under 10% hydrogen at 300 °C for 4 h. The sample was then vacuum dried at 200 °C for 12 h and the process repeated, giving a Pt loading of 0.5 wt% as determined by Inductively Coupled Plasma Optical Emission Spectrometry (ICP-OES).

### Catalytic hydrogenolysis

Hydrogenolysis experiments were performed in 100 ml Parr autoclaves (Series 4590 Micro Reactor) equipped with an overhead stirrer and thermocouple. 500 mg Pt/STO catalyst and ∼5 g polyethylene (Sigma Aldrich, *M*_w_ = 4000 g/mol, *M*_n_ = 1700 g/mol) were placed in a glass sleeve inside the reactor. The autoclave was sealed and flushed with Ar and ultra-high purity (UHP) H_2_ ten times each. The reactor was heated to 300 °C, agitated with an impeller at 800 rpm, and charged with H_2_ to 170 psi. After 72 h, the reactor was cooled to room temperature and vented to the atmosphere. The waxy product between the walls of the reactor and the walls of the glass sleeve was collected, filtered hot through silica gel (Davisil Grade 646, Millipore Sigma), washed with n-hexane, and dried under vacuum at ∼55 °C.

### High-temperature gel-permeation chromatography (HT-GPC)

The molecular weights (*M*_n_ and *M*_w_) of the starting polymer and hydrogenolysis products were determined by High-Temperature Gel-Permeation Chromatography (HT-GPC) on an Agilent PL220 equipped with refractive index (RI) and viscometer detectors with three Agilent PL-Gel Mixed B columns and one PL-Gel Mixed B guard column. Monodisperse polyethylene standards (∼300 g/mol to 120 g/mol) were used to build the calibration curve. The eluent was 1,2,4-trichlorobenzene (TCB) containing 0.01 wt% 3,5-di-tert-butyl-4-hydroxytoluene (BHT) at a flow rate of 1.0 ml/min at 150 °C. The samples were prepared in TCB at a concentration of ∼1.0 to ∼4.0 mg/ml and heated at 150 °C for 24 h prior to injection.

### Nuclear magnetic resonance (NMR) spectroscopy

Solution-phase nuclear magnetic resonance (NMR) spectra were collected using a Bruker Avance III 500 MHz NMR spectrometer (11.7 T) at 120 °C in 1,1,2,2-tetrachloroethane-*d*_2_ and referenced internally to residual solvent signals. ^1^H NMR spectra (500 MHz) were recorded with 32 scans and ^13^C{^1^H} NMR spectra (125 MHz) with ∼9500 scans. Spectra were analyzed using MestReNova (v11.0.1, Mestrelab Research S.L.). The branching density was determined by ^1^H NMR spectroscopy according to: # branches per 1000 C = (CH_3_/3)/((CH + CH_2_ + CH_3_)/2) × 1000 where CH_3_, CH_2_, and CH refer to the integrations obtained for methyl, methylene, and methine resonances, respectively.

## Preparation of depolymerized polyethylene sample in culture flasks

### Bolus

The bolus preparation was performed by measuring out centimeter-scale pieces of the depolymerized polyethylene sample and dropping them into culture flasks containing media.

### Coated

Coating preparation was performed by heating flasks on a hot plate to approximately 65 °C. A bolus of sample was then added and the flask was gently rotated and titled to form a coating on the bottom of the flask that had no gaps in coverage over its entire area.

### Imperfect coating

Imperfect coatings were generated by first performing the same procedure used for coating preparation. Upon cooling, the coating was then manually agitated with a sterile laboratory scoop to form gaps in the coating coverage. Chunks of sample that were scraped off through the process were added back into the flask as small boluses.

### Floating disk

Flasks with media already added, but not cells, were heated on a hot plate to approximately 65 °C. A bolus of sample was then added and allowed to melt into an oily, floating liquid layer. Upon cooling, the solidified sample resembled a thin, floating plate which was subsequently broken into smaller chunks with a pipette tip. All different sample preparation methods were performed under sterile conditions.

### Supplementary Information

**Additional file 1: Table S1.** Strains, plasmids, and primers used in this study. **Figure S1.** A5 4mer recombinant silk protein primary sequence. **Figure S2.** Scatter plots of the cfu/ml of *P.* *aeruginosa* RR1 over time when hexadecane is the sole carbon source. **Figure S3.** Scatter plots of the cfu/ml of *P.* *oleovorans* over time when hexadecane is the sole carbon source. **Figure S4.** Recombinant green fluorescent protein (GFPuv) production in LB media using the RK2-GFPuv plasmid. **Figure S5.** Growth and plasmid maintenance for *P. aeruginosa *RR1 RK2-GFPuv and *P. oleovorans *RK2-GFPuv in 0.46% w/v hexadecane with 5 g/L NH_4_Cl. **Figure S6.** Growth and plasmid maintenance for *P. aeruginosa *RR1 B5-GFPuv and *P. aeruginosa *RR1 RSF-GFPuv in 0.46% w/v hexadecane with 1.9 g/L NH_4_NO_3_. **Figure S7.** Agarose gel after colony PCR on colonies of *P. aeruginosa *RR1 g-GFPuv and wild-type *P. aeruginosa *RR1. **Figure S8.** Recombinant green fluorescent protein (GFPuv) production in LB media using strain *P. aeruginosa *RR1 g-GFPuv (genomic integration of GFPuv gene). **Figure S9.** Exemplary lysates of P. aeruginosa RR1 g-GFPuv exposed to UV light when grown on 0.46% w/v C16 with 5 g/L NH_4_Cl (top) or 4.6% w/v C16 with 5 g/L NH_4_Cl (bottom) and exposed to various induction times. **Figure S10.** Expression cultures of *P. aeruginosa* RR1 g-A5 grown on 0.46% w/v hexadecane with 5 g/L NH_4_Cl. **Figure S11.** The depolymerized polyethylene sample. **Figure S12.** Different morphologies of the depolymerized polyethylene sample tested for growth of strain *P. aeruginosa *RR1 g-A5. **Figure S13.** Expression cultures of *P. aeruginosa *RR1 g-A5 grown using depolymerized polyethylene as the sole carbon source. **Figure S14.** SDS PAGE after nickel chromatography showing the A5 4mer silk protein purified from lysates of *P. aeruginosa *RR1 g-A5 that were grown using depolymerized polyethylene as the sole carbon source.

## Data Availability

The datasets used and/or analysed during the current study are available from the corresponding author on reasonable request.
