# Supplementary material for: Two-step conversion of polyethylene into recombinant proteins using a microbial platform
Source: Microb Cell Fact. 2023 Oct 17;22:214. doi: 10.1186/s12934-023-02220-0 (PMC10580613; doi:10.1186/s12934-023-02220-0)
Supplement: Supplementary file 1 — Additional file 1: Table S1. Strains, plasmids, and primers used in this study. Figure S1. A5 4mer recombinant silk protein primary sequence. Figure S2. Scatter plots of the cfu/ml of P. aeruginosa RR1 over time when hexadecane is the sole carbon source. Figure S3. Scatter plots of the cfu/ml of P. oleovorans over time when hexadecane is the sole carbon source. Figure S4. Recombinant green fluorescent protein (GFPuv) production in LB media using the RK2-GFPuv plasmid. Figure S5. Growth and plasmid maintenance for P. aeruginosa RR1 RK2-GFPuv and P. oleovorans RK2-GFPuv in 0.46% w/v hexadecane with 5 g/L NH4Cl. Figure S6. Growth and plasmid maintenance for P. aeruginosa RR1 B5-GFPuv and P. aeruginosa RR1 RSF-GFPuv in 0.46% w/v hexadecane with 1.9 g/L NH4NO3. Figure S7. Agarose gel after colony PCR on colonies of P. aeruginosa RR1 g-GFPuv and wild-type P. aeruginosa RR1. Figure S8. Recombinant green fluorescent protein (GFPuv) production in LB media using strain P. aeruginosa RR1 g-GFPuv (genomic integration of GFPuv gene). Figure S9. Exemplary lysates of P. aeruginosa RR1 g-GFPuv exposed to UV light when grown on 0.46% w/v C16 with 5 g/L NH4Cl (top) or 4.6% w/v C16 with 5 g/L NH4Cl (bottom) and exposed to various induction times. Figure S10. Expression cultures of P. aeruginosa RR1 g-A5 grown on 0.46% w/v hexadecane with 5 g/L NH4Cl. Figure S11. The depolymerized polyethylene sample. Figure S12. Different morphologies of the depolymerized polyethylene sample tested for growth of strain P. aeruginosa RR1 g-A5. Figure S13. Expression cultures of P. aeruginosa RR1 g-A5 grown using depolymerized polyethylene as the sole carbon source. Figure S14. SDS PAGE after nickel chromatography showing the A5 4mer silk protein purified from lysates of P. aeruginosa RR1 g-A5 that were grown using depolymerized polyethylene as the sole carbon source. [file 12934_2023_2220_MOESM1_ESM.docx]

**Supplementary Materials**

**Two-Step Conversion of Polyethylene into Recombinant Proteins Using a Microbial Platform**

Alexander Connor ^1,a,b^, Jessica Lamb^c^, Massimiliano Delferro^c^, Mattheos Koffas ^a,b,^*, R. Helen Zha ^a,b,^*

^a^ Department of Chemical and Biological Engineering, Rensselaer Polytechnic Institute, Troy, NY, 12180, USA

^b^ Center for Biotechnology and Interdisciplinary Studies, Rensselaer Polytechnic Institute, Troy, NY, 12180, USA

^c^ Argonne National Laboratory, 9700 S Cass Ave, Lemont, IL 60439, USA

* Corresponding Author emails: [koffam@rpi.edu](mailto:koffam@rpi.edu) , [zhar@rpi.edu](mailto:zhar@rpi.edu)

**Supplementary Tables and Figures Contents**

**Supplementary Table S1:** Strains, plasmids, and primers used in this study.

**Supplementary Figure S1:** A5 4mer recombinant silk protein primary sequence

**Supplementary Figure S2:** Scatter plots of the cfu/ml of *P. aeruginosa* RR1 over time when hexadecane is the sole carbon source.

**Supplementary Figure S3:** Scatter plots of the cfu/ml of *P. oleovorans* over time when hexadecane is the sole carbon source.

**Supplementary Figure S4:** Recombinant green fluorescent protein (GFPuv) production in LB media using the RK2-GFPuv plasmid.

**Supplementary Figure S5:** Growth and plasmid maintenance for *P. aeruginosa* RR1 RK2-GFPuv and *P. oleovorans* RK2-GFPuv in 0.46% w/v hexadecane with 5 g/L NH_4_Cl.

**Supplementary Figure S6:** Growth and plasmid maintenance for *P. aeruginosa* RR1 B5-GFPuv and *P. aeruginosa* RR1 RSF-GFPuv in 0.46% w/v hexadecane with 1.9 g/L NH_4_NO_3_.

**Supplementary Figure S7:** Agarose gel after colony PCR on colonies of *P. aeruginosa* RR1 g-GFPuv and wild-type *P. aeruginosa* RR1.

**Supplementary Figure S8:** Recombinant green fluorescent protein (GFPuv) production in LB media using strain *P. aeruginosa* RR1 g-GFPuv (genomic integration of GFPuv gene).

**Supplementary Figure S9:** Exemplary lysates of P. aeruginosa RR1 g-GFPuv exposed to UV light when grown on 0.46% w/v C16 with 5 g/L NH4Cl (top) or 4.6% w/v C16 with 5 g/L NH4Cl (bottom) and exposed to various induction times.

**Supplementary Figure S10:** Expression cultures of *P. aeruginosa* RR1 g-A5 grown on 0.46% w/v hexadecane with 5 g/L NH_4_Cl.

**Supplementary Figure S11:** The depolymerized polyethylene sample.

**Supplementary Figure S12:** Different morphologies of the depolymerized polyethylene sample tested for growth of strain *P. aeruginosa* RR1 g-A5.

**Supplementary Figure S13:** Expression cultures of *P. aeruginosa* RR1 g-A5 grown using depolymerized polyethylene as the sole carbon source.

**Supplementary Figure S14:** SDS PAGE after nickel chromatography showing the A5 4mer silk protein purified from lysates of *P. aeruginosa* RR1 g-A5 that were grown using depolymerized polyethylene as the sole carbon source.

**Supplementary Table 1: Strains, plasmids, and primers**

| **Strains** | | | | |
| --- | --- | --- | --- | --- |
| **Strain** | | **Description** | **Source/Accession No.** | |
| *P. aeruginosa* RR1 | | Wild-type strain | NCIMB 14928 | |
| *P. oleovorans* | | Wild-type strain | NRRL B-14682 | |
| *P. aeruginosa* RR1 RK2-GFPuv | | *P. aeruginosa* RR1, transformed with pBb(RK2)1k-GFPuv | This work | |
| *P. aeruginosa* RR1 B5-GFPuv | | *P. aeruginosa* RR1, transformed with pBb(B5)1k-GFPuv | This work | |
| *P. aeruginosa* RR1 RSF-GFPuv | | *P. aeruginosa* RR1, transformed with pBb(RSF1010)1k-GFPuv | This work | |
| *P. oleovorans* RK2-GFPuv | | *P. oleovorans* transformed with pBb(RK2)1k-GFPuv | This work | |
| *P. aeruginosa* RR1 g-GFPuv | | *P. aeruginosa* RR1 with genomically integrated GFPuv gene | This work | |
| *P. aeruginosa* RR1 g-A5 | | *P. aeruginosa* RR1 with genomically integrated A5 4mer silk gene | This work | |
| **Plasmids** | | | | |
| **Plasmid** | | **Description** | **Source/Accession No.** | |
| pBb(RK2)1k-GFPuv | | IPTG inducible GFPuv expression, low copy number plasmid | Addgene #106394  [29] | |
| pBb(B5)1k-GFPuv | | IPTG inducible GFPuv expression, high copy number plasmid | Addgene #106393  [29] | |
| pBb(RSF1010)1k-GFPuv | | IPTG inducible GFPuv expression, moderate copy number plasmid | Addgene #106395  [29] | |
| pTNS2 | | Tn7 transposase expression, for genomic integration at *att*Tn*7* site | Addgene #64968  [30] | |
| pUC18-mini-Tn7T-LAC | | For genomic integration at *att*Tn*7* site, includes tac promoter and lac operator | Addgene #64965  [30] | |
| pUC18-Tn7T-GFPuv | | pUC18-mini-Tn7T-LAC with GFPuv gene | This work | |
| pUC18-Tn7T-A5 | | pUC18-mini-Tn7T-LAC with A5 4mer silk protein gene | This work | |
| pET19b-A5 | | pET19b with A5 4mer silk gene | [47] | |
| pBb(RK2)1k-A5 | | pBb(RK2)1k backbone with A5 4mer gene | This work | |
| **Primers** | | | | |
| **Primer** | **Sequence** | | | **Source** |
| glmS-down | 5′ GCACATCGGCGACGTGCTCTC 3′ | | | [30] |
| glmS-up | 5′ CTGTGCGACTGCTGGAGCTGA 3′ | | | [30] |
| GFPuv-Gf | 5’ CGCGCCACTAGTCAGAATTCAAAAGATCTTTTAAGAAGGAG 3’ | | | This work |
| GFPuv-Gr | 5’ CGCGCGAAGCTTCCTTGTGGGGTCAGTTCC 3’ | | | This work |
| A5-19bf | 5’ CGCGCCAGATCTTTTAAGAAGGAGATATACCATGGGCCATCA 3’ | | | This work |
| A5-19br | 5’ CCGTGTCTCGAGTTAGGATCCACTAGTTCCGGAGC 3’ | | | This work |
| A5-Gf | 5’ CGCGCCGAGCTCCAGAATTCAAAAGATCTTTTAAGAAGGAG 3’ | | | This work |

**A5 4mer Primary Sequence:**

MGHHHHHHHHHHSSGHIDDDDKHMLEHMPG

**GPGQQ AAAAA GPGQQGPGQQGPGQQGPGEQGPGSG**

**GPGQQ AAAAA GPGQQGPGQQGPGQQGPGEQGPGSG**

**GPGQQ AAAAA GPGQQGPGQQGPGQQGPGEQGPGSG**

**GPGQQ AAAAA GPGQQGPGQQGPGQQGPGEQGPGSG**

TSGS

**Supplementary Figure S1:** A5 4mer recombinant silk protein primary sequence**.** The primary sequence was designed to mimic the natural structure of major ampullate two dragline silk (MaSp2) from orb-weaving spiders^47^. The sequence contains a 10x histidine tag for purification as well as four tandem repeats of a 35 amino acid segment (bolded) comprised primarily of GPGQQ repeats adjacent to a single segment of five tandem alanine residues. The molecular weight is 16.1 kDa.

**Supplementary Figure S2:** Scatter plots of the cfu/ml of *P. aeruginosa* RR1 over time when using hexadecane as the sole carbon source under a variety of culture conditions. Hexadecane (C16) concentrations are provided as % w/v. Nitrogen sources include ammonium chloride (NH_4_Cl) at 2 or 5 g/L or ammonium nitrate (NH_4_NO_3_) at 1.9 or 3.8 g/L. Data points were derived from the average of three replicates, with cfu/ml measured at 0, 7, 24, 33, 48, 55, 72, 79, and 96 hours post inoculation.

**Supplementary Figure S3:** Scatter plots of the cfu/ml of *P. oleovorans* over time when using hexadecane as the sole carbon source under a variety of culture conditions. Hexadecane (C16) concentrations are provided as % w/v. Nitrogen sources include ammonium chloride (NH_4_Cl) at 2 or 5 g/L or ammonium nitrate (NH_4_NO_3_) at 1.9 or 3.8 g/L. Data points were derived from the average of three replicates, with cfu/ml measured at 0, 7, 24, 33, 48, 55, 72, 79, and 96 hours post inoculation.

**a b c d**


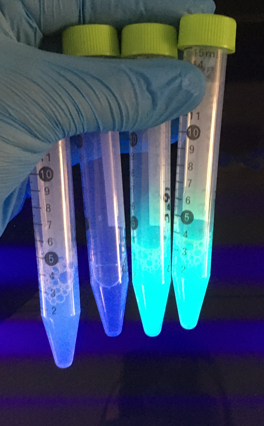


**Supplementary Figure S4:** Recombinant green fluorescent protein (GFPuv) production in LB media using the RK2-GFPuv plasmid. Lysates exposed to UV light of **(a)** *P. aeruginosa* RR1 RK2-GFPuv uninduced **(b)** *P. oleovorans* RK2-GFPuv uninduced **(c)** *P. aeruginosa* RR1 RK2-GFPuv induced with 0.3 mM IPTG **(d)** *P. oleovorans* RK2-GFPuv induced with 0.3 mM IPTG.

**Supplementary Figure S5:** Growth and plasmid maintenance for *P. aeruginosa* RR1 RK2-GFPuv and *P. oleovorans* RK2-GFPuv in 0.46% w/v hexadecane with 5 g/L NH_4_Cl. **a)** Growth of *P. aeruginosa* RR1 RK2-GFPuv at 24 and 48 hours post inoculation with and without kanamycin supplementation. **b)** *P. aeruginosa* RR1 RK2-GFPuv plasmid maintenance at 24 and 48 hours post inoculation in the presence of 50 µg/ml kanamycin. **c)** Growth of *P. oleovorans* RK2-GFPuv at 24 and 48 hours post inoculation with and without kanamycin supplementation. **d)** *P. oleovorans* RK2-GFPuv plasmid maintenance at 24 and 48 hours post inoculation in the presence of 50 µg/ml kanamycin. Error bars represent standard deviations from the mean values of three replicates.


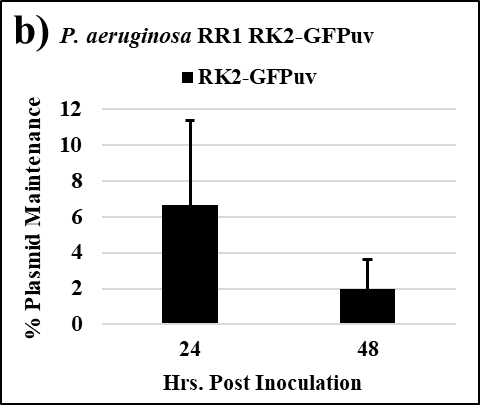

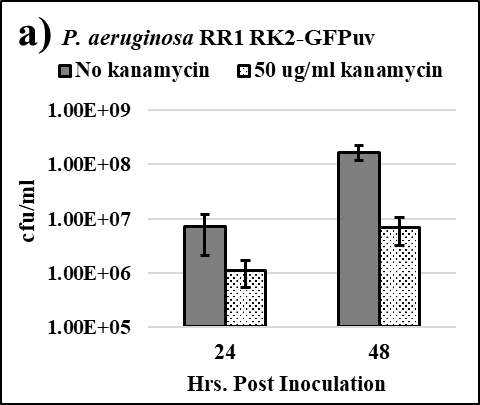

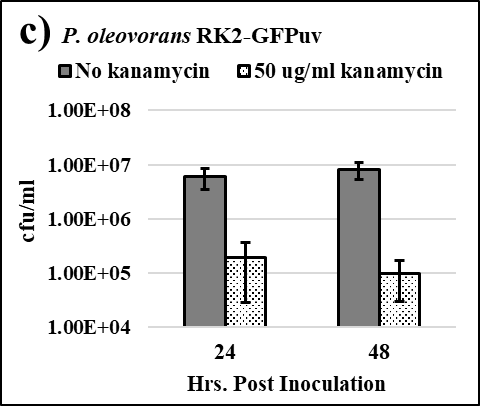

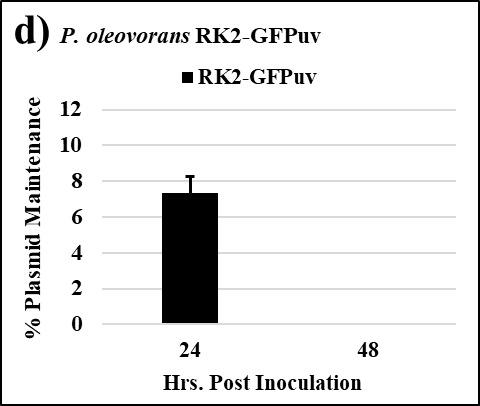


**Supplementary Figure S6:** Growth and plasmid maintenance for *P. aeruginosa* RR1 B5-GFPuv and *P. aeruginosa* RR1 RSF-GFPuv in 0.46% w/v hexadecane with 1.9 g/L NH_4_NO_3_. **a)** Growth of *P. aeruginosa* RR1 B5-GFPuv at 24 and 48 hours post inoculation with and without kanamycin supplementation. **b)** *P. aeruginosa* RR1 B5-GFPuv plasmid maintenance at 24 and 48 hours post inoculation in the presence of 50 µg/ml kanamycin. **c)** Growth of *P. aeruginosa* RR1 RSF-GFPuv at 24 and 48 hours post inoculation with and without kanamycin supplementation. **d)** *P. aeruginosa* RR1 RSF-GFPuv plasmid maintenance at 24 and 48 hours post inoculation in the presence of 50 µg/ml kanamycin. Error bars represent standard deviations from the mean values of three replicates.


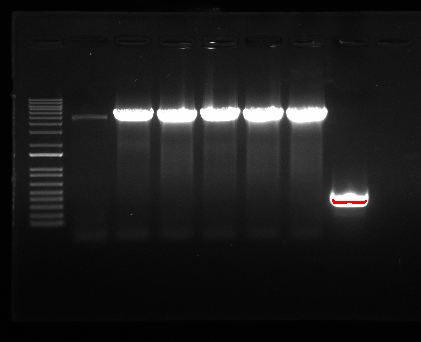


**0.3 kb--**

**5 kb---**

**Supplementary Figure S7:** Agarose gel after colony PCR on colonies of *P. aeruginosa* RR1 g-GFPuv and wild-type *P. aeruginosa* RR1. Lanes (left to right): DNA ladder, six colonies of *P. aeruginosa* RR1 g-GFPuv, and a single colony of wild-type *P. aeruginosa* RR. Colonies of *P. aeruginosa* RR1 g-GFPuv properly integrated with the GFPuv construct show amplification of a 5 kb band. Wild-type *P. aeruginosa* RR1 shows amplification of a 0.3 kb band^30^.

**a b c**


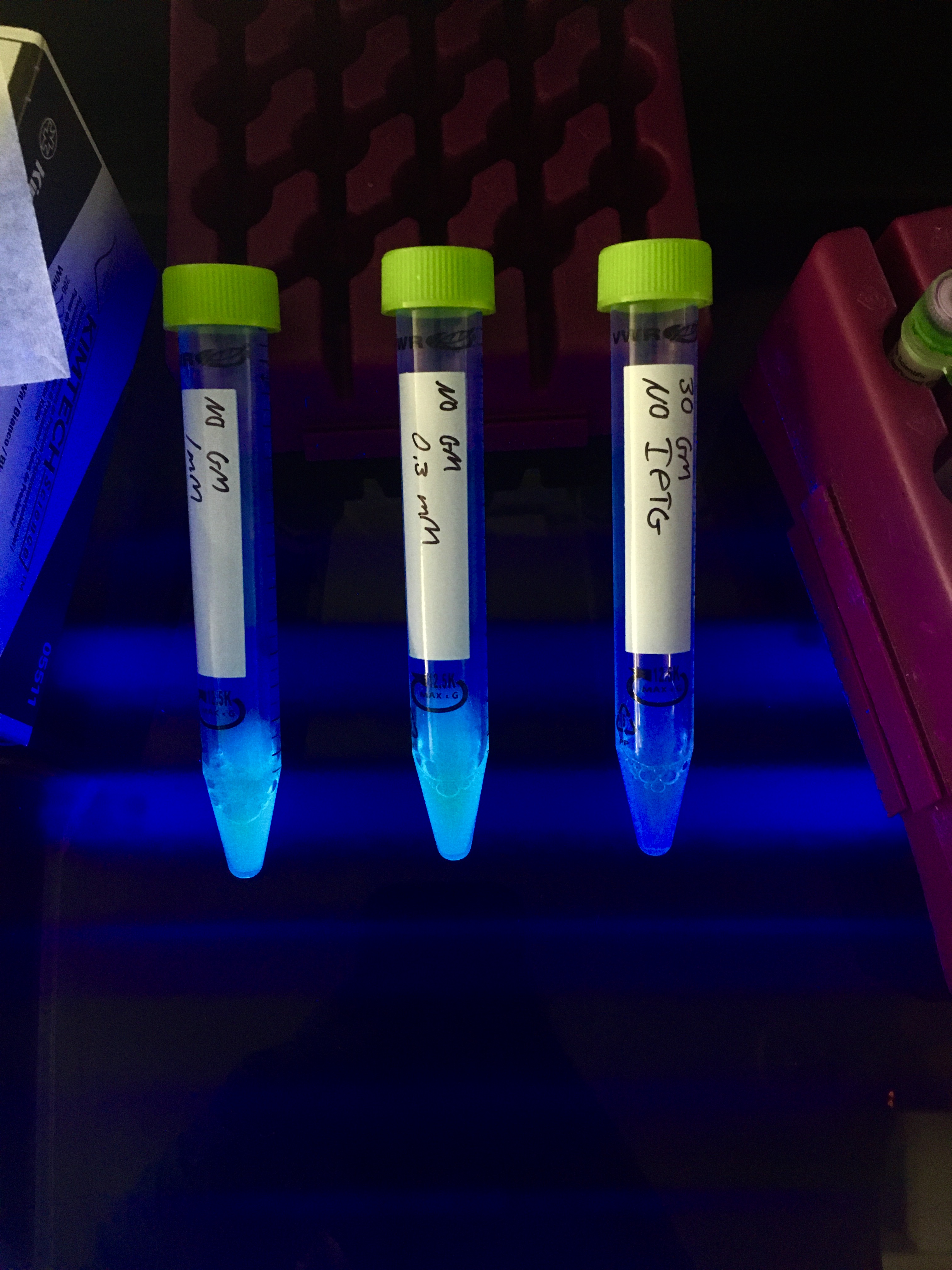


**Supplementary Figure S8:** Recombinant green fluorescent protein (GFPuv) production in LB media using strain *P. aeruginosa* RR1 g-GFPuv (genomic integration of GFPuv gene). Lysates exposed to UV light of **(a)** *P. aeruginosa* RR1 g-GFPuv induced with 1 mM IPTG **(b)** *P. aeruginosa* RR1 g-GFPuv induced with 0.3 mM IPTG **(c)** *P. aeruginosa* RR1 g-GFPuv uninduced. All strains cultures were grown in the liquid LB media without gentamicin supplementation.

**Supplementary Figure S9**: Exemplary lysates of P. aeruginosa RR1 g-GFPuv exposed to UV light when grown on 0.46% w/v C16 with 5 g/L NH4Cl (top) or 4.6% w/v C16 with 5 g/L NH4Cl (bottom) and exposed to various induction times. Cultures were either uninduced (none) or induced with 0.3 mM IPTG at 24 (@24 hrs), 48 (@48 hrs), or 72 (@72 hrs) hours post inoculation, corresponding to 72-, 48-, or 24-hour expression times, respectively. The time at which IPTG is added may affect the intensity of visible GFPuv production and was found to be dependent upon culture conditions and corresponded to the measured GFPuv titer. IPTG added at any of the three tested times resulted in relatively indistinguishable visual GFPuv production when cultures were grown using 0.46% w/v hexadecane and 5 g/L NH4Cl (top image). However, a decreasing intensity of visual GFPuv production can be seen for cultures grown on 4.6% C16 with 5 g/L NH4Cl as the time at induction progresses from 24, to 48, to 72 hours. The pale blue color present for uninduced cultures (none) stems from production of a natural pyocyanin pigment^43^.


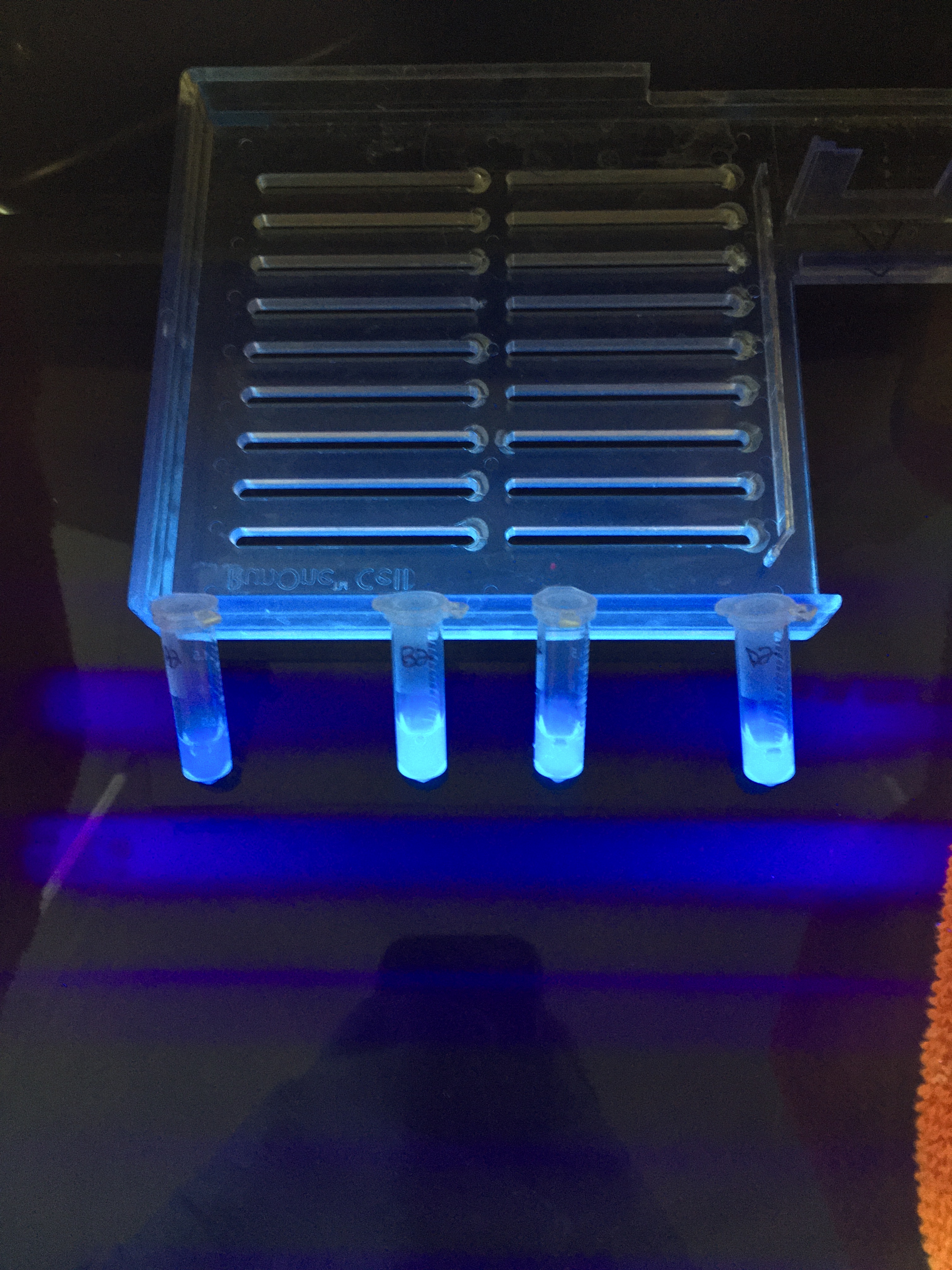

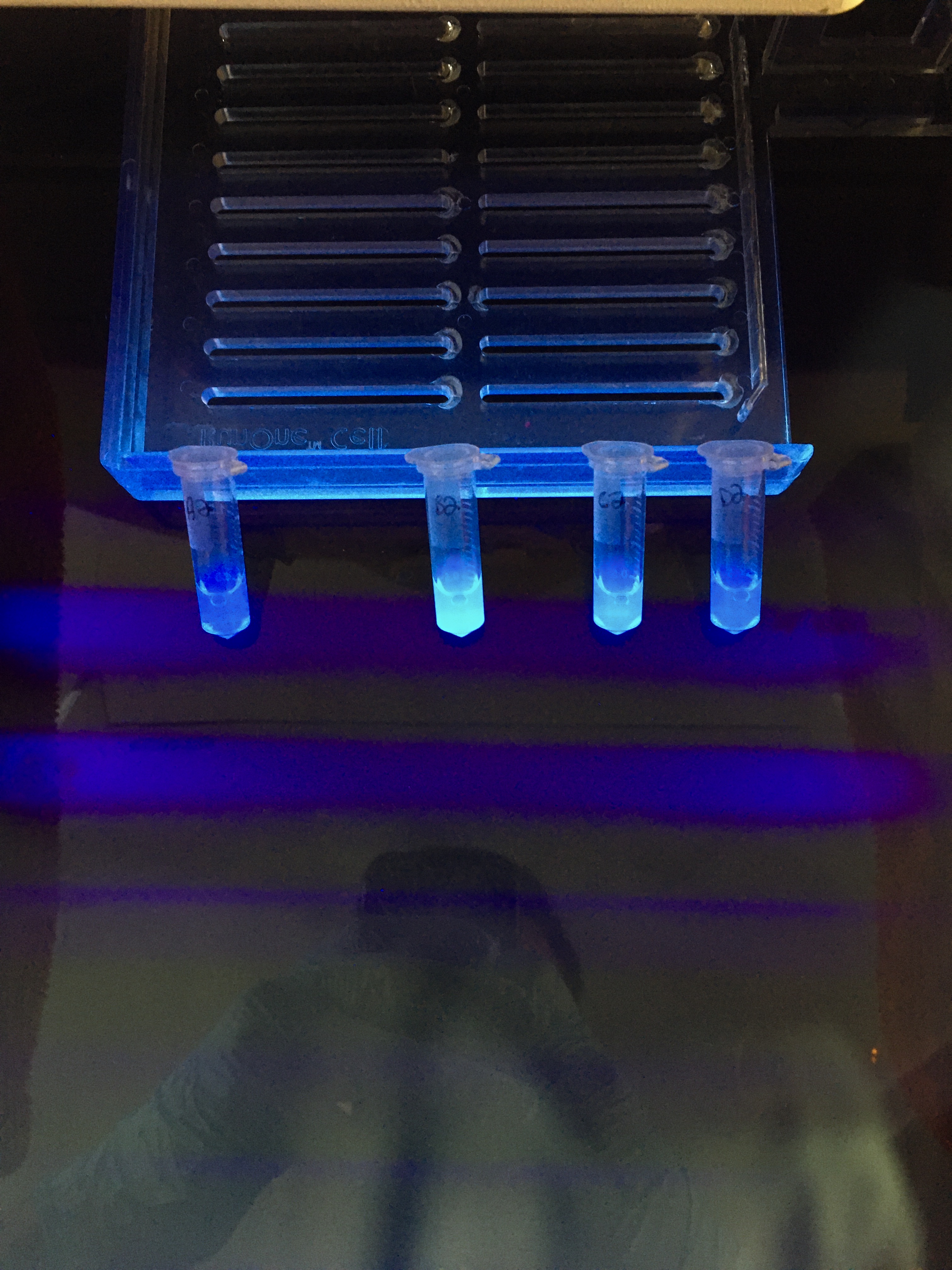


None @24 hrs @48 hrs @72 hrs


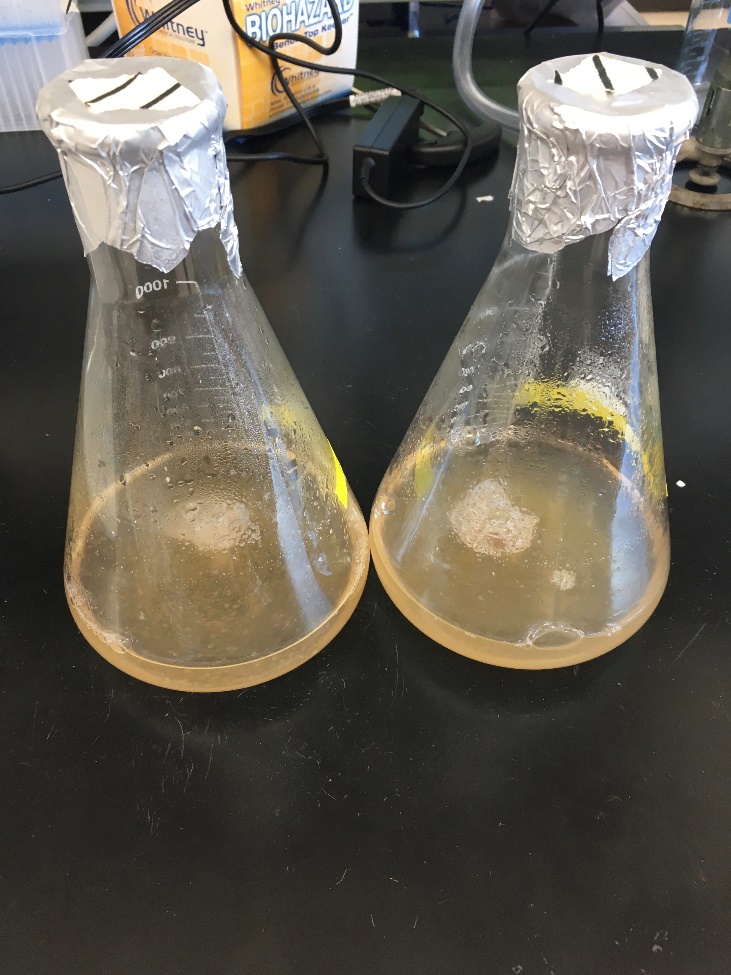
**
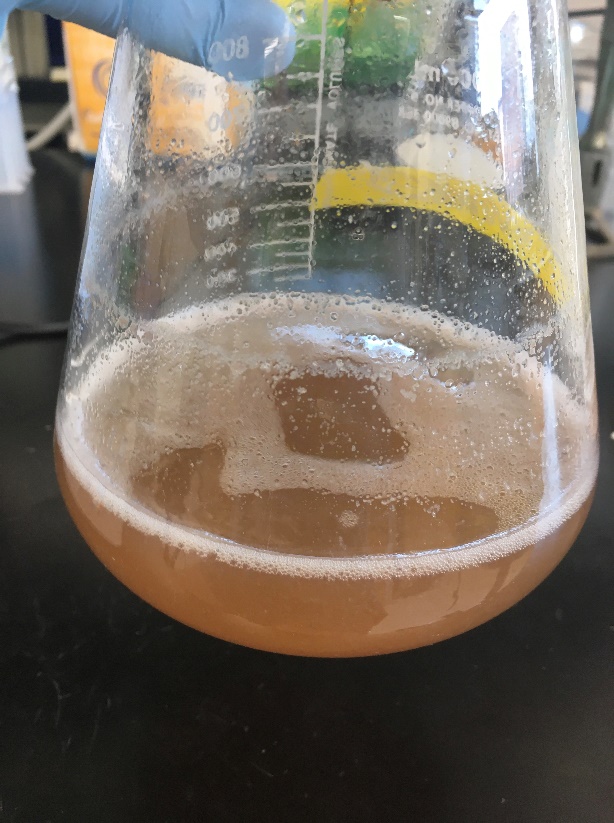
**

**Supplementary Figure S10:** Expression cultures of *P. aeruginosa* RR1 g-A5 grown on 0.46% w/v hexadecane with 5 g/L NH_4_Cl at 48 hours post inoculation (left image, two flasks) and 72 hours post inoculation and induction (right image, single flask).


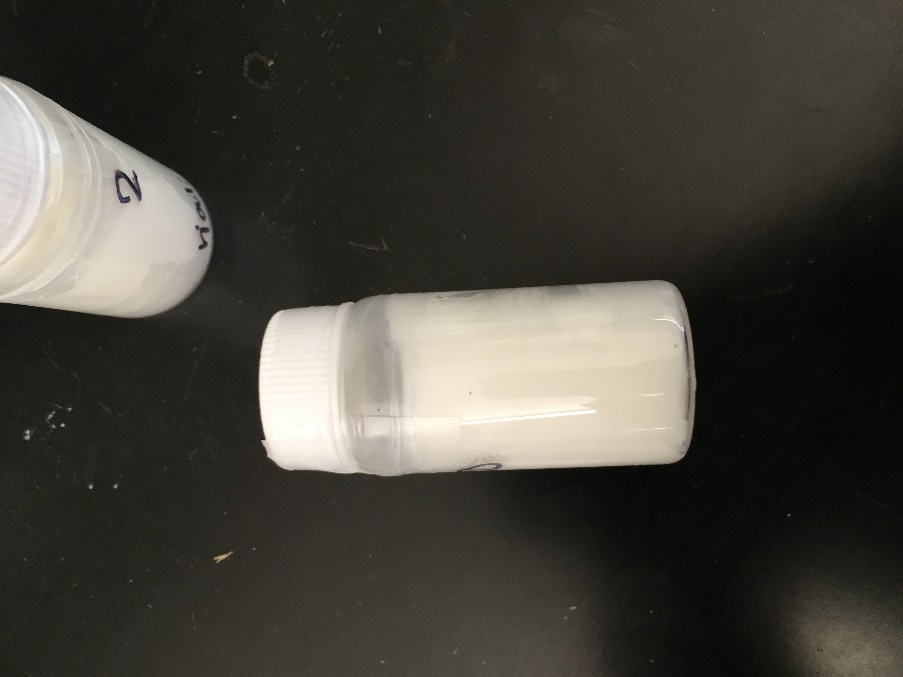


**Supplementary Figure S11:** The depolymerized polyethylene sample. The sample contains a distribution of alkanes (Mw = 306 g/mol, Mn = 242 g/mol, and Mw/Mn = 1.26) and a non-negligible degree of branching (N_branch/1000C_ = 111). The sample is white, cohesive, and has the consistency of a soft wax.

**
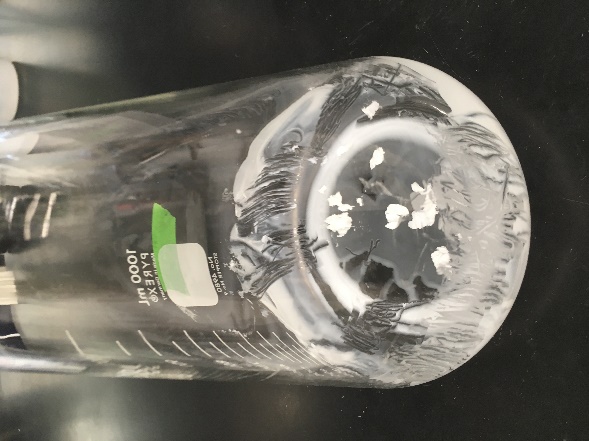

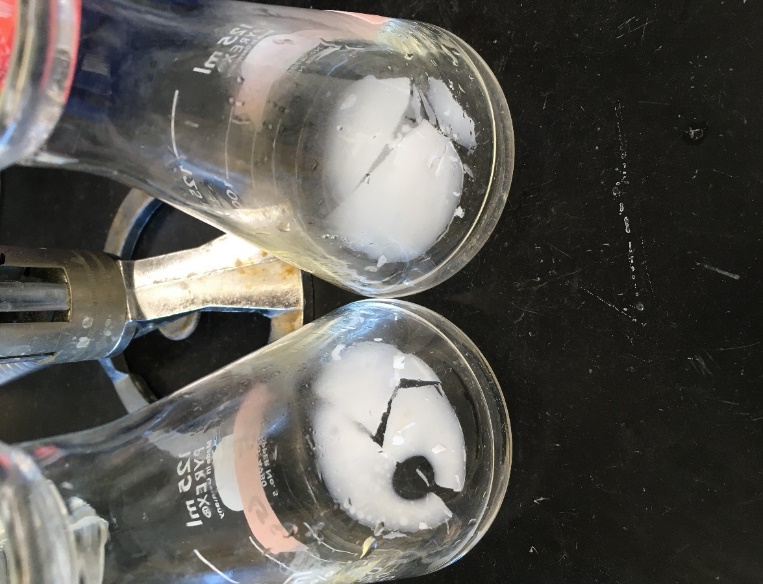
**
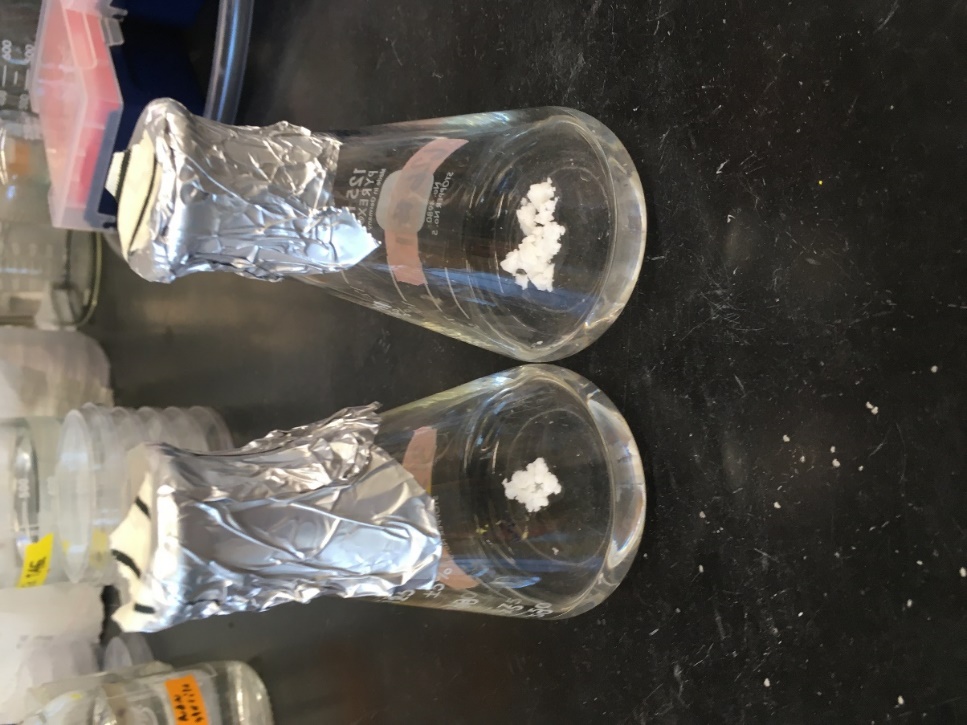
**
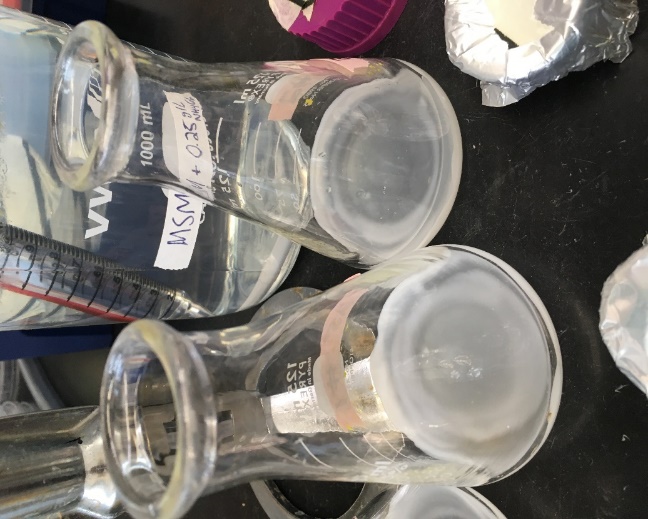
**

**a**

**b**

**d**

**c**

**Supplementary Figure S12:** Different morphologies of the depolymerized polyethylene sample tested for growth of strain *P. aeruginosa* RR1 g-A5. All pictures were taken prior to inoculation. **a)** **Bolus preparation.** Small masses of sample were added to flasks at 12.5 g/L (left) and 25 g/L (right). Using this method, the sample floats on top of media and individual chunks adhere to each other. **b)** **Coating preparation.** The sample was added to the bottom of flasks at 25 g/L (left) and 12.5 g/L (right) before being gently melted on a hot plate with swirling of the flask to create a coating. The coatings contained no gaps in coverage of the flask within their total area. **c)** **Floating disk preparation.** A bolus of sample was added to heated, sterile media, resulting in a top layer of liquid sample. Upon cooling, the sample solidified and resembled a thin, floating plate which could be subsequently broken into smaller chunks with a pipette tip. **d) Imperfect coating preparation.** Coatings were prepared as in **(b)**, upon solidification the coating was agitated to create imperfect coating coverage. Sections of coating that were removed were added back into the flask as small boluses to maintain consistent per liter concentrations of the sample.

**
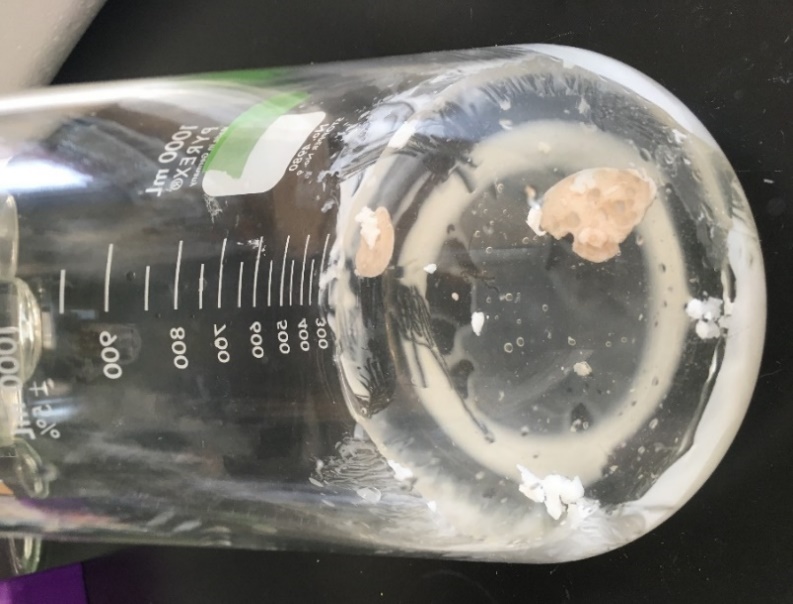

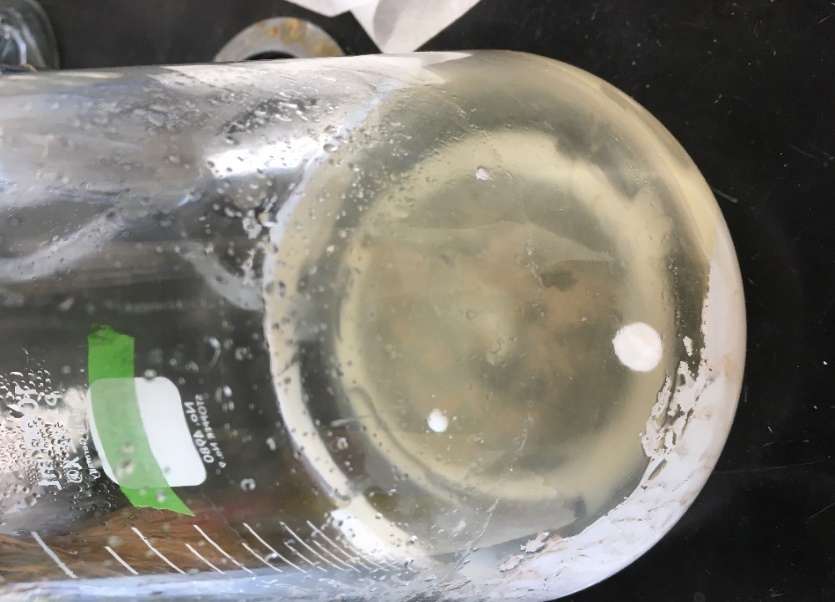
**

**b**

**a**

**
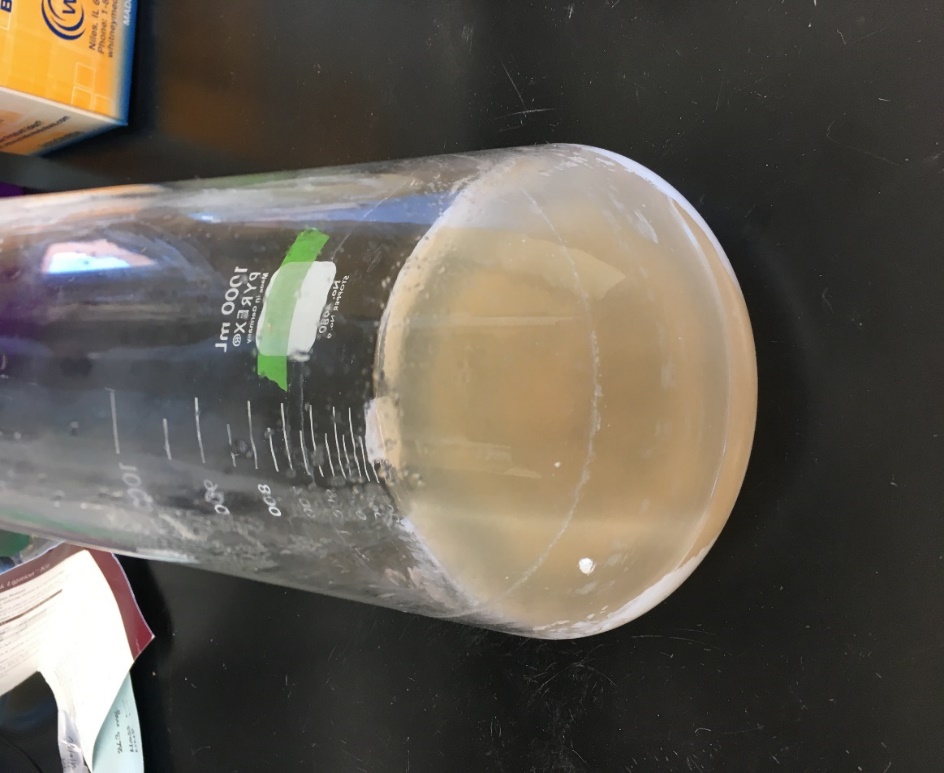

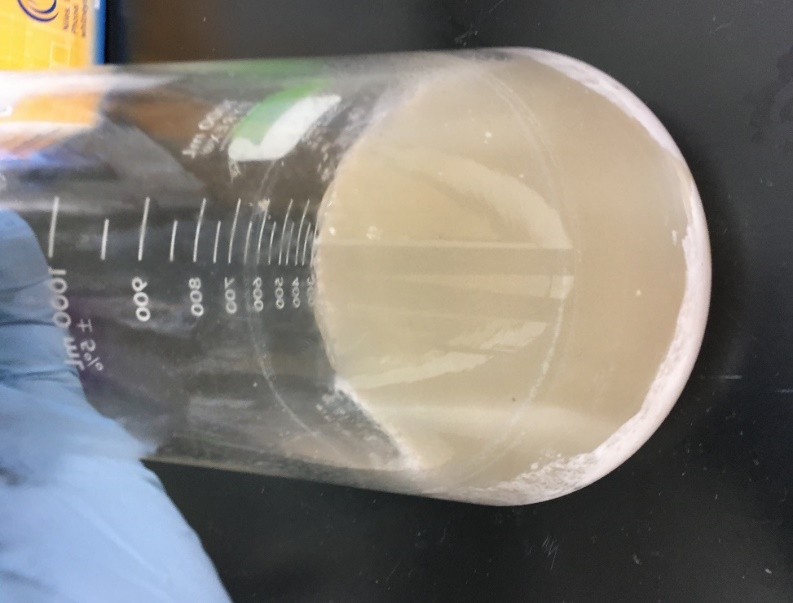
**

**d**

**c**

**Supplementary figure S13:** Expression cultures of *P. aeruginosa* RR1 g-A5 grown using 5 g/L NH_4_Cl and 12.5 g/L of the depolymerized polyethylene as the sole carbon source and prepared using the “imperfect coating” method. **a)** time of inoculation and induction with 1 mM IPTG **b)** 24 hours post inoculation **c)** 48 hours post inoculation **d)** 72 hours post inoculation (time of harvest).


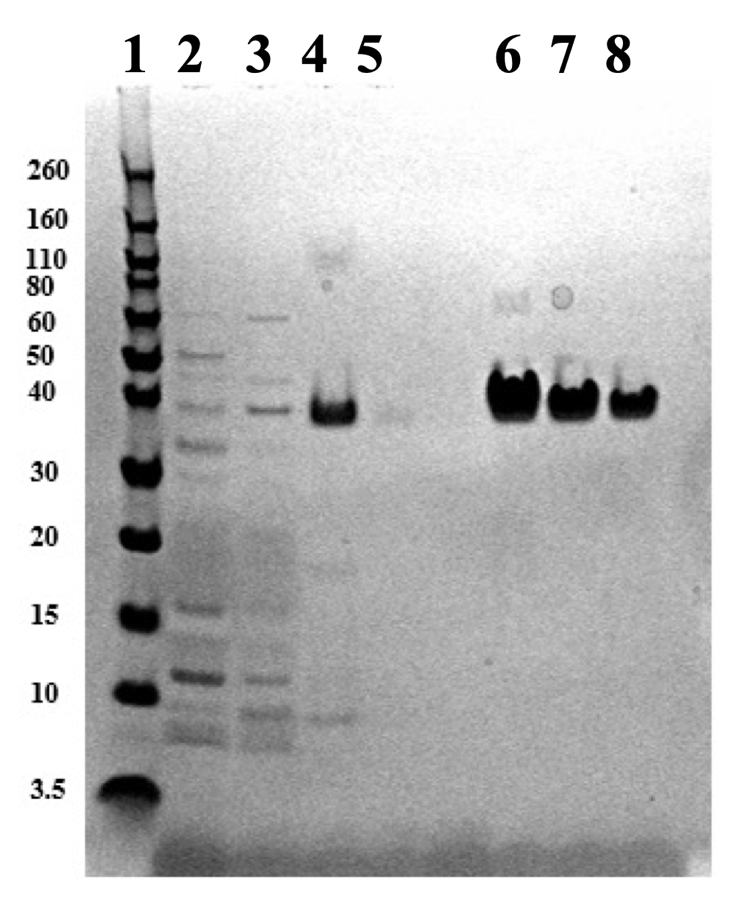


**Supplementary Figure S14:** SDS PAGE after nickel chromatography showing the A5 4mer silk protein purified from lysates of *P. aeruginosa* RR1 g-A5 that were grown using depolymerized polyethylene as the sole carbon source (12.5 g/L depolymerized polyethylene prepared with the “imperfect coating” method and supplemented with 5 g/L NH_4_Cl). Lanes (1) Protein ladder, with mass listed to left in kDa (2) flow through (3) wash (4) first elution fraction (5) second elution fraction (6-8) standards of A5 4mer protein, 2, 1, and 0.5 mg/L. The band in lane 4 (elution) is shown at an identical molecular weight (~38 kDa) as standards of the A5 4mer protein produced in *E. coli* (lanes 5-7). As previously documented, the 16 kDa A5 4mer silk protein appears at ~38 kDa due to its high level of structural disorder and subsequent aberrant mobility through SDS PAGE^47^.
